# Quinate-based ligands for irreversible inactivation of the bacterial virulence factor DHQ1 enzyme—A molecular insight^†^

**DOI:** 10.3389/fmolb.2023.1111598

**Published:** 2023-01-24

**Authors:** Ángela Rodríguez, María Maneiro, Emilio Lence, José M. Otero, Mark J. van Raaij, Paul Thompson, Alastair R. Hawkins, Concepción González-Bello

**Affiliations:** ^1^ Centro Singular de Investigación en Química Biolóxica e Materiais Moleculares (CiQUS), Departamento de Química Orgánica, Universidade de Santiago de Compostela, Santiago de Compostela, Spain; ^2^ Departamento de Estructura de Macromoléculas, Centro Nacional de Biotecnología (CSIC), Madrid, Spain; ^3^ Newcastle University Biosciences Institute, The Medical School, Newcastle University, Newcastle upon Tyne, United Kingdom

**Keywords:** enzyme recognition, biomacromolecule simulations, irreversible inhibition, binding mode, organic synthesis, ligand activation, type I dehydroquinase, anti-virulence agents

## Abstract

Irreversible inhibition of the enzyme type I dehydroquinase (DHQ1), a promising target for anti-virulence drug development, has been explored by enhancing the electrophilicity of specific positions of the ligand towards covalent lysine modification. For ligand design, we made use of the advantages offered by the intrinsic acid-base properties of the amino substituents introduced in the quinate scaffold, namely compounds **6**–**7** (*R* configuration at C3), to generate a potential leaving group, as well as the recognition pattern of the enzyme. The reactivity of the C2–C3 bond (Re face) in the scaffold was also explored using compound **8**. The results of the present study show that replacement of the C3 hydroxy group of (–)-quinic acid by a hydroxyamino substituent (compound **6**) provides a time-dependent irreversible inhibitor, while compound **7**, in which the latter functionality was substituted by an amino group, and the introduction of an oxirane ring at C2–C3 bond, compound **8**, do not allow covalent modification of the enzyme. These outcomes were supported by resolution of the crystal structures of DHQ1 from *Staphylococcus aureus* (*Sa*-DHQ1) and *Salmonella typhi* (*St*-DHQ1) chemically modified by **6** at a resolution of 1.65 and 1.90 Å, respectively, and of *St*-DHQ1 in the complex with **8** (1.55 Å). The combination of these structural studies with extensive molecular dynamics simulation studies allowed us to understand the molecular basis of the type of inhibition observed. This study is a good example of the importance of achieving the correct geometry between the reactive center of the ligand (electrophile) and the enzyme nucleophile (lysine residue) to allow selective covalent modification. The outcomes obtained with the hydroxyamino derivative **6** also open up new possibilities in the design of irreversible inhibitors based on the use of amino substituents.

## 1 Introduction

The mechanism of action of most antibiotics in clinical use is based on inactivation of enzymes/proteins encoded by essential genes, thus preventing the synthesis and assembly of key components that are needed for bacterial growth (viability) (Cook and Wright 2022). The main metabolic pathways targeted by these drugs include cell-wall biosynthesis, DNA replication, RNA transcription, folate biosynthesis, and protein biosynthesis. Although this strategy is very effective and has given rise to a good arsenal of “life-saving” compounds, it causes substantial stress in the bacteria, which usually triggers the appearance of antibiotic-resistant strains. As such, to reduce the emergence and worldwide spread of multidrug-resistant bacteria, an innovative antibacterial approach that is increasingly being explored comprises inhibition of the bacterial capacity to produce the infection (pathogenesis) (Theuretzbacher and Piddock 2019; Hotinger et al., 2021; Krell and Matilla 2022). This “antibiotic-free” approach has several advantages over the traditional strategies dealing with bacterial viability, including: i) pathogenic bacteria are specifically targeted, with little or no impact on the normal human microbiota; ii) less selective pressure may be imposed to the development of bacterial resistance; and iii) natural clearing by the host immune system as a result of the *in vivo* scenario created, which resembles vaccination with a live attenuated strain (Dickey et al., 2017).

**FIGURE 1:**
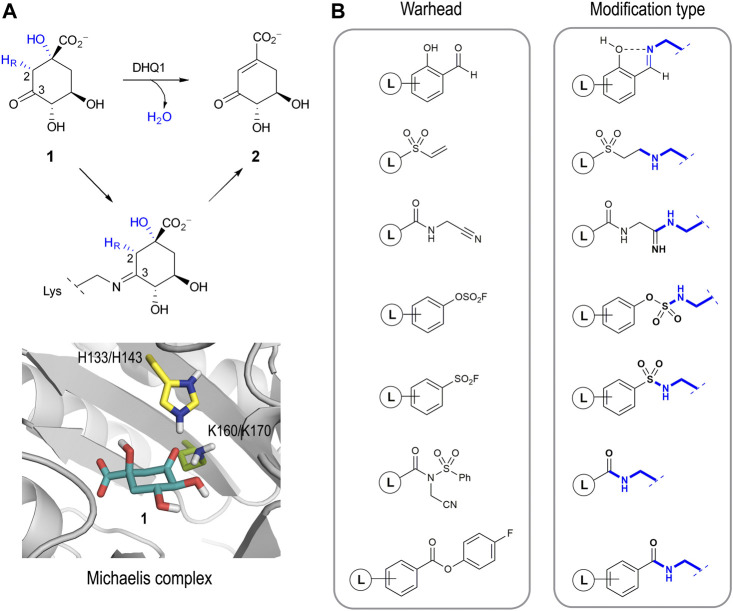
**(A)** Overall *syn* elimination of water from 3-dehydroquinic acid (**1**) catalyzed by DHQ1. The enzymatic process involves the formation of diverse Schiff base species *via* a catalytic lysine residue. Magnified view of the Michaelis complex obtained by MD simulation studies. Only the catalytic side-chain residues are shown. **(B)** Relevant reported electrophilic warheads for reversible/irreversible targeting of lysine residues and the covalent modification obtained.

Several studies have identified the enzyme type I dehydroquinase (DHQ1, 3-dehydroquinate dehydratase, EC 4.2.1.10) as a promising target for anti-virulence drug development. The interesting features of this enzyme that have attracted our attention are: i) DHQ1 acts as a virulence factor *in vivo* as deletion of the *aroD* gene, which codes for this enzyme, has proven to afford satisfactory live oral vaccines (Tacket et al., 1992; Tacket et al., 1992; Kärnell et al., 1993; Dilts et al., 2000; Tacket and Levine 2007; Malcova et al., 2009; Revolledo and Ferreira 2012; Racz et al., 2013; Cunningham et al., 2020); ii) the *aroD* mutation is auxotrophic, affects bacterial cell wall integrity and blocks the bacterial ability to form biofilms (Cano et al., 2003; Nógrády et al., 2003; Sebkova et al., 2008); iii) DHQ1 is upregulated in small colony variants, a pathogenic form of bacteria that facilitates persistent and recurrent infections (Proctor et al., 2006; Zhang et al., 2017); iv) no mammalian homologues of the DHQ1 enzyme have been identified; and v) DHQ1 is present in several pathogenic bacteria, including *Escherichia coli, Salmonella typhi* and *Staphylococcus aureus*. DHQ1 catalyzes the reversible dehydratation of 3-dehydroquinic acid (**1**) to 3-dehydroshikimic acid (**2**), which is the third step in the shikimic acid pathway (Figure 1A) (González-Bello 2015). This conversion implies the overall *syn* elimination of water and removal of the pro-*R* hydrogen at C2 in **1**. It follows a covalent catalysis mechanism with the formation of transient Schiff base species´ between the ketone moiety of the substrate and the ɛ-amino group of a catalytic lysine residue (K160/K170 in *Sa*-DHQ1 and *St*-DHQ1, respectively), which are finally hydrolyzed to regenerate the enzyme. An essential histidine residue (H133/H143 in *Sa*-DHQ1 and *St*-DHQ1, respectively), is also involved in the formation/hydrolysis of the Schiff base intermediates (Leech et al., 1995; Leech et al., 1998).

The development of selective irreversible inhibitors is envisaged as an attractive approach for the inhibition of enzymes undergoing covalent catalysis as there is no dissociation equilibrium for competitive strategies. These types of inhibitors have additional benefits compared with non-covalent ligands, especially improved potency and prolonged drug effects (permanent inactivation of the target), and they are usually more robust against pharmacokinetic issues (Singh et al., 2011; Bauer 2015; Zhang et al., 2019). After years of overestimating their possible side effects, this type of inhibitor has become particularly important in drug-discovery programs, especially for non-chronic therapies such as those against infectious diseases and cancer (Sanderson 2013). The development of a selective irreversible inhibitor is not an easy task, because the introduction of overly reactive electrophiles in the scaffold will increase the chances of having undesired off-target effects. To overcome this issue, a booming strategy involves the use of barely reactive electrophilic groups that are only activated towards reaction upon binding to their target. In these cases, the correct arrangement of the ligand and the suitable geometry of the electrophilic group in the ligand relative to the enzyme/protein nucleophile are key for the success of the covalent modification. In recent years, the design of ligands capable of selectively modifying lysine residues is probably one of the most pursued and challenging goals (Hoyt et al., 2019). Although these residues are very abundant in proteins, in most cases they are not nucleophiles as the ɛ-amino group is protonated at physiological pH. As such, an ability to target protein pockets comprising residues that are able to generate transitory “reactive lysine residues” is crucial for this approach. Good examples of electrophilic warheads for reversible and irreversible covalent modification of lysine residues include salicylaldehyde-based probes (Yang et al., 2022), vinyl sulfones (Anscombe et al., 2015), nitriles (Smith et al., 2018), aryl fluorosulfates (Mortenson et al., 2018), aromatic sulfonyl fluorides (Grimster et al., 2013; Narayanan and Jones 2015; Zhao et al., 2017), *N*-acyl-*N*-alkyl sulfonamides (Tamura et al., 2018), and 4-fluorobenzoic esters (Dalton et al., 2018), amongst others (Figure 1B). In these examples, although the electrophilic character of the warhead is enhanced upon ligand binding, the reaction occurs at sp^2^/sp carbon atoms *per se* activated to a certain extent. An ability to get the reaction to occur at carbon atoms of the ligand with little electrophilic character (sp^3^), which are very common, appears a challenging task that has been little explored to date but which might expand our options for drug design.

**FIGURE 2:**
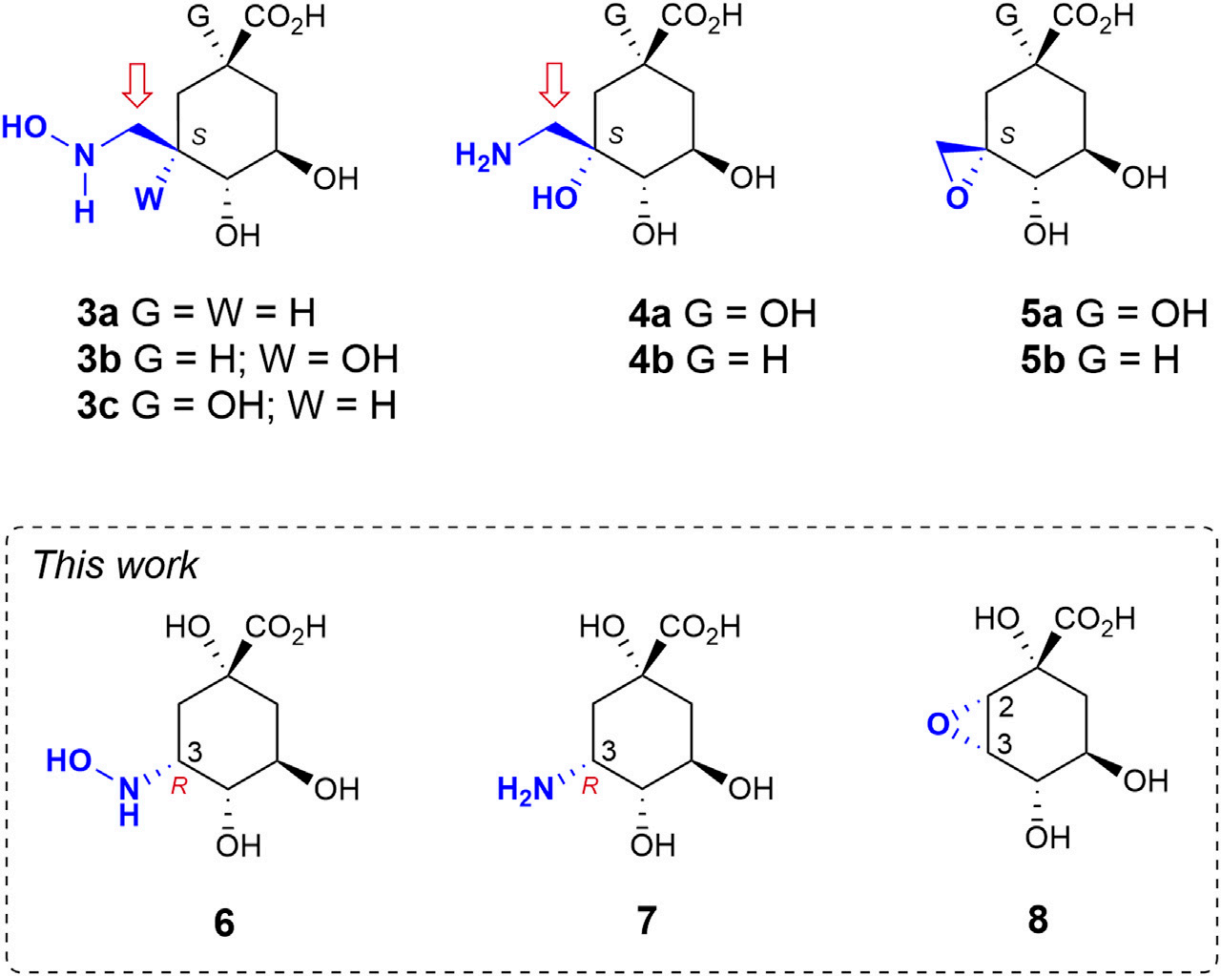
Relevant reported irreversible inhibitors and targeted compounds. The methylene group undergoing lysine modification is indicated with an arrow. The numbering corresponds to the natural substrate.

Herein we study the possible activation of cyclic sp3-hybridized carbons for irreversible inhibition of the target. To this end, we make use of the intrinsic acid-base properties of amino substituents to generate a leaving group, as well as the recognition pattern of the targeted enzyme pocket. We reported recently that compounds **3** bearing a hydroxyaminomethyl group with an *S* configuration at C3 in the natural substrate (compound **1**) are irreversible inhibitors of the enzyme DHQ1 (Figure 2) (Maneiro et al., 2019). A direct nucleophilic attack by the ɛ-amino group of the catalytic lysine at the exocyclic methylene group would take place with the formation of an amine linkage. We have also shown that the introduction of an aminomethyl and hydroxy substituent with an *S* configuration at C3 in **1** (compounds **4**) also results in covalent modification of the catalytic lysine residue to afford a stable Schiff base as they are converted into reactive epoxides **5** (González-Bello et al., 2015; Lence et al., 2020). Herein we report the synthesis of compounds **6**–**7** in which enhancement of the electrophilicity of an endocyclic carbon atom in the ligand towards covalent modification is attempted by direct introduction of the amino functionality into the quinate scaffold (Figure 2). These compounds are substrate analogs in which the carbonyl group at C3 has been replaced by a hydroxyamino and an amino group with *R* configuration (compounds **6** and **7**, respectively). The numbering of the natural substrate has been maintained for clarity. This stereochemistry was selected based on preliminary computational studies on the Michaelis complex of the DHQ1 enzymes from *S. typhi* (*St*-DHQ1) and *S. aureus* (*Sa*-DHQ1), which showed that the ketone group at C3 is activated by the catalytic histidine residue *via* its Re face towards nucleophilic attack by the catalytic lysine residue from the opposite site (Figure 1A). In addition, the reactivity of C3 in the quinate scaffold was also explored by introduction of an oxirane moiety with an *S,S* configuration at the C2-C3 bond (compound **8**) to compare the geometry requirements of the C3 position towards nucleophilic attack. The crystal structures of *Sa*-DHQ1 and *St*-DHQ1 enzymes chemically modified by **6** at a resolution of 1.65 and 1.90 Å, respectively, and of *St*-DHQ1 in a complex with epoxide **8** (1.55 Å) are provided. These structural studies, in combination with various biochemical and molecular dynamics (MD) simulation studies, allowed us to understand the molecular basis of the type of inhibition observed.

## 2 Results and discussion

### 2.1 Synthesis of compounds 6–7

**SCHEME 1:**
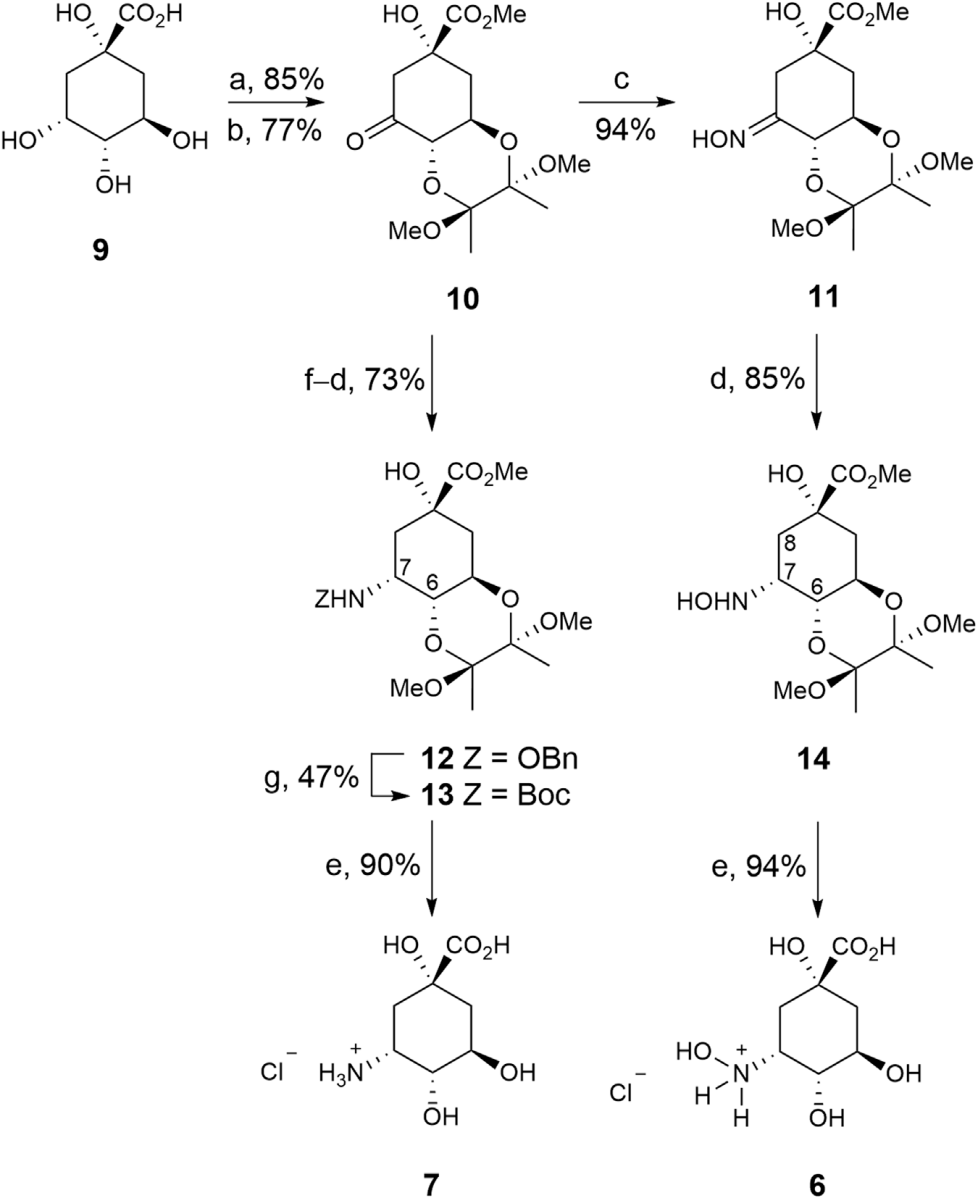
Synthesis of compounds **6**–**7**. *Reagents and conditions.* (a) (MeCO)_2_, CH(OMe)_3_, MeOH, camphorsulfonic acid, 65°C. (b) PDC, MS 4 Å, CH_2_Cl_2_, RT. (c) NH_2_OH.HCl, NaOAc, MeCN, H_2_O, RT. (d) NaBH_3_CN, MeOH, HCl, RT. (e) HCl (0.3 M), 100°C. (f) NH_2_OBn.HCl, NaOAc, MeOH, MS 4 Å, RT. (g) 1. H_2_(g), Pd/C (10%), AcOH, MeOH, RT. 2. Boc_2_O, Et_3_N, DMF, RT.

The strategy used to synthesise compounds **6**–**7** involved the initial preparation of ketone **10**, which was synthesized in two steps from commercially available (–)-quinic acid (**9**) using previously reported protocols (Scheme 1) (Toscano et al., 2003). Treatment of ketone **10** with hydroxylamine gave oxime **11**, in 89% yield, which was converted into compound **14** by regioselective reduction of the imino group with sodium cyanoborohydride (85% yield). The stereochemistry of the resulting new chiral center was determined by NOE experiments. Thus, irradiation of the H8_ax_ signal (1.78 ppm) in compound **14** led to enhancement of the H6 and H7 signals by 6.2% and 4.2%, respectively. Finally, hydrolysis of the ester group with concomitant removal of the protecting groups by heating at 100°C with diluted HCl afforded the desired 3-hydroxyaminoquinic acid (**6**), as its hydrochloride salt, in 94% yield.

3-Aminoquinic acid (**7**) was prepared by regioselective reductive amination of ketone **10** with benzylhydroxylamine to afford compound **12** in 73% yield. The benzyl group in **12** was removed by hydrogenolysis under acidic conditions and the resulting amine was converted into the *Boc*-amino protected derivative **13** by treatment with di-*tert*-butyl dicarbonate to facilitate its purification (47% yield). NOE experiments also confirmed the *S* configuration of the new chiral center as irradiation of the H7 signal (4.16 ppm) in **13** led to enhancement of the H6 and NH signals by 5.2% and 1.3%, respectively. Finally, compound **13** was transformed into the target compound **7**, as its hydrochloride salt, in a similar manner to compound **6** from **14**.

### 2.2 Synthesis of compound 8

**SCHEME 2:**
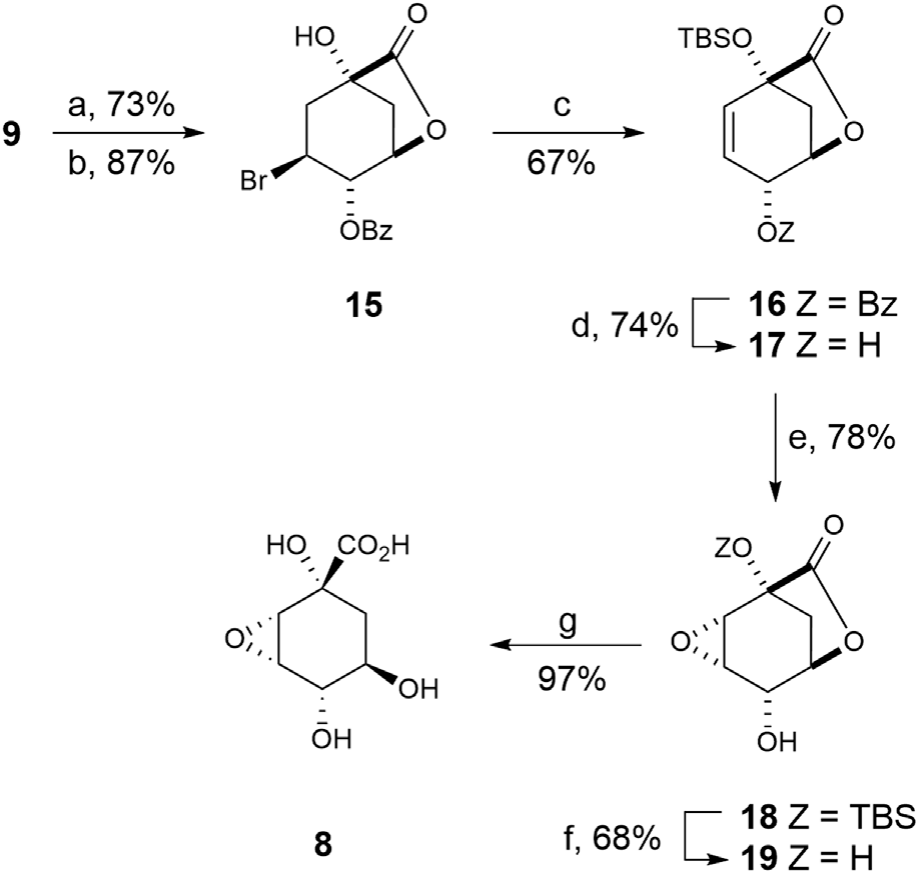
Synthesis of compound **8**. *Reagents and conditions.* (a) PhCHO, DMF, PhMe, *p*-TsOH (cat), ∆. (b) NBS, AIBN (cat), PhH, ∆. (c) DBU, ClTBS, CH_3_CN, ∆. (d) 1. KCN, MeOH, RT. 2. NaH, THF, 0°C. (e) MCPBA, NaHCO_3_, CH_2_Cl_2_, ∆. (f) (20:1) TFA/H_2_O, RT. (g) 1. LiOH, RT, 2. Amberlite IR-120.

This compound was prepared from previously reported epoxide **18**, which is synthesized in seven steps from (–)-quinic acid **9**) (Scheme 2) (González et al., 2003). Briefly, quadruple protection of (–)-quinic acid (**9**) by reaction with benzaldehyde in acid medium, followed by treatment with *N*-bromosuccinimide in the presence of AIBN as radical initiator afforded bromobenzoate **15** (Bartlett et al., 1986). Treatment of **15** with *tert*-butyldimethylsilyl chloride and excess of DBU led to simultaneous formation of the silylether and the double bond. Treatment of benzoate **16** with potassium cyanide in methanol afforded hydroxycarbolactone **17** and the corresponding methyl ester, which was converted into **17** with sodium hydride. Epoxidation of allylic alcohol **17** by treatment with *meta*-chloroperbenzoic acid in the presence of sodium bicarbonate afforded the epoxy alcohol **18** as the sole diasteroisomer. Removal of the TBS-protecting group in **18** by treatment with aqueous TFA gave alcohol **19** in 68% yield. Finally, basic hydrolysis of lactone **19** and subsequent protonation with Amberlite IR-120 (H^+^) gave the desired oxirane derivative **8** in 97% yield.

### 2.3 Inhibitory studies

The inhibitory capacity of compounds **6**–**8** against DHQ1 enzymes from *S. aureus* and *S. typhi* was evaluated. For irreversible inhibition assays, the enzyme was incubated with the ligand and its remaining activity was progressively determined using aliquots from the incubation samples and the control. For reversible competitive inhibition assays, the activity of the enzyme in the presence of increasing amount of ligand was evaluated. The enzyme activity was determined by monitoring the increase in absorbance at 234 nm in the UV spectrum due to formation of the enone-carboxylate chromophore of the enzyme product, 3-dehydroshikimic acid **2**). Compound **6** was found to be a time-dependent irreversible inhibitor of the two enzymes with *K*_I_ values of 90 ± 8 and 130 ± 12 µM and *k*_inact_ values of 8 ± 1 and 13 ± 1 ms^−1^ against *Sa*-DHQ1 and *St*-DHQ1, respectively (Supplementary Figure S1). In contrast, compounds **7** and **8** were found to be reversible competitive inhibitors of the two enzymes. For amino derivative **7**, IC_50_ values of 482 ± 19 and 528 ± 33 µM against *Sa*-DHQ1 and *St*-DHQ1, respectively, were obtained. The inhibitory capacity of compound **8** was found to be poor, with IC_50_ values > 2 mM for both enzymes.

### 2.4 Structural studies

To obtain structural information on the binding mechanism of compounds **6**–**8**, and further details of the covalent modification caused by **6**, crystallization of DHQ1 from both *S. aureus* and *S. typhi* with the ligands reported herein was attempted*.* As a result, protein crystals with the appropriate diffracting properties complexed with compounds **6** and **8** were obtained. For epoxide **8**, crystals were obtained by soaking using apo-*St*-DHQ1 crystals, while for hydroxylamine **6** they were achieved by co-crystallization. Crystals were mounted into cryoloops and were directly flash frozen by rapid immersion in liquid nitrogen. The crystallization conditions contained a sufficient amount of cryo-protectant (PEG) to directly freeze the protein crystals in liquid nitrogen without further manipulation. X-ray diffraction data were collected from crystals of *Sa*-DHQ1/**6**, *St*-DHQ1/**6** and *St*-DHQ1/**8** cryo-cooled in a stream of cold nitrogen gas (100 K) at ambient pressure using synchrotron radiation. The structures were determined by molecular replacement and refined. Full details are provided in the experimental section. A summary of the statistics following data reduction and processing is given in Table 1.

**TABLE 1:** Crystallographic data collection and refinement statistics for the *St*-DHQ1/**6** **and *Sa*-DHQ1/**6** **enzyme adducts and *St*-DHQ1/** **8** **enzyme complex** *^**a**^***.

Data processing^a^	*St*-DHQ1/6	*Sa*-DHQ1/6	*St*-DHQ1/8
Space group	*P*1	*P*121	*P*22121
Cell parameters:			
a, b, c Å)	42.50, 43.63, 72.37	36.38, 79.57, 83.54	42.71, 47.08, 105.20
α, β, γ (◦)	84.25, 85.96, 60.94	90, 101.17, 90	90, 90, 90
Wavelength Å)	0.97926	0.97926	1.00556
Observed reflections*^b^*	58418 (8,189)*^c^*	184526 (27589)*^c^*	116502 (12643)*^c^*
Resolution range Å)	71.99–1.90 (2.00–1.90)	81.95–1.65 (1.74–1.65)	105.20–1.55 (1.63–1.55)
Wilson B (Å^2^)	21.5	25.8	13.4
Multiplicity	1.8 (1.7)	3.4 (3.5)	4.1 (3.8)
Completeness (%)	93.5 (91.4)	98.0 (97.7)	89.4 (73.4)
Mean (I)/sd(I))	6.7 (1.9)	19.1 (3.4)	22.6 (3.4)
Mn(I) half-set correlation CC(1/2)	0.0 (0.0)	1.00 (0.95)	1.00 (0.90)
*R*_merge_	0.056 (0.277)	0.027 (0.323)	0.036 (0.398)
Refinement*^d^*			
Resolution range Å)	38.05–1.90 (1.93–1.90)	79.70–1.65 (1.67–1.65)	52.60–1.55 (1.57–1.55)
Reflections used in refinement*^c^*	31164 (2,179)	52133 (3775)	26630 (1,541)
Reflections used for R_free_	1,638 (119)	2,631 (185)	1,299 (91)
*R* factor*^e^*	0.218 (0.470)	0.219 (0.290)	0.167 (0.220)
*R*_free_ *^f^*	0.278 (0.440)	0.255 (0.320)	0.200 (0.240)
rmsd (bonds Å)/angles (°))	0.010/1.6	0.008/1.5	0.013/1.8
Final model			
Protein/inhibitor/water/sodium/sulfate/chloride/HEPES atoms	3886/22/144/4/0/0/0	3733/22/150/0/10/1/0	1960/13/128/0/10/0/15
Average B protein/inhibitor/water/sodium/sulfate/chloride/HEPES (Å^2^)	36.9/38.0/34.5/55.0/0/0/0	34.9/35.5/34.1/0/72.8/35.9/0	16.9/17.0/23.3/0/35.7/0/27.7
Ramachandran statistics*^g^* (%)	97.4/100.0	98.7/100.0	97.2/100.0
PDB ID	8B2B	8B2A	8B2C

^a^ Results from SCALA (Evans 2006).

^b^ No sigma cut-off or other restrictions were used for inclusion of reflections.

^c^ Values in parentheses are for the highest resolution bin, where applicable.

^d^ Results from REFMAC5 (Kovalevskiy et al., 2018).

^e^ *R*-factor = Σ ||*Fobs*(*hkl*)|—|*Fcalc(hkl)*||/Σ |*Fobs(hkl)*|.

^f^ According to Brünger (Brünger 1997).

^g^ According to the program MOLPROBITY (Williams et al., 2018). The percentages indicated are for residues in favored and total allowed regions, respectively.

#### 2.4.1 DHQ1/6 enzyme adducts

The two adducts *Sa-*DHQ1/**6** and *St-*DHQ1/**6** crystallized as dimers with two molecules in the asymmetric unit (namely chains A and B) and were solved at a resolution of 1.65 and 1.90 Å, respectively. The two crystallographically independent copies superimpose well onto each other, with root-mean-square (rms) differences of 0.363 Å (*Sa*-DHQ1) and 0.117 Å (*St*-DHQ1) after superposition of C^α^-atom pairs. Both chains are virtually identical.

**FIGURE 3:**
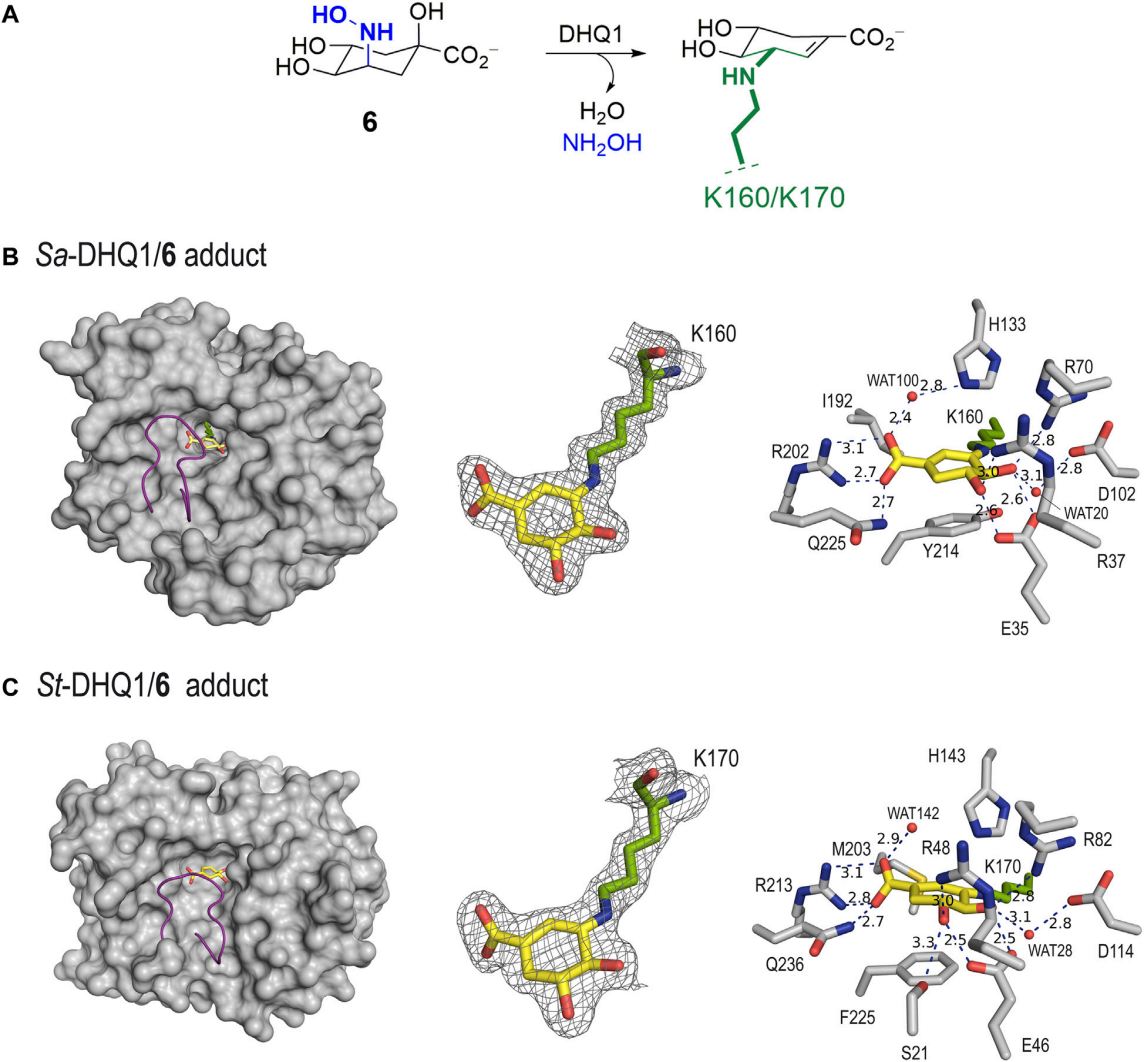
**(A)** Schematic representation of the chemical modification to the catalytic lysine residue of *Sa*-DHQ1 and *St*-DHQ1, as identified by X-ray crystallography. **(B,C)** Crystal structures of *Sa*-DHQ1 (B, PDB ID 8B2A, 1.65 Å, chain A) and *St*-DHQ1 (C, PDB ID 8B2B, 1.90 Å, chain A) covalently modified by hydroxylamine **6**. Overall views of the enzyme adducts and interactions of the modified inhibitor (yellow) are shown. The substrate-covering loop (purple) is shown as a cartoon to visualize the active site. Hydrogen-bonding and electrostatic interactions between the ligands and both homologous enzymes are shown as dashed lines (blue). Relevant residues are shown and labeled. Unbiased electron density for inhibitor **6** and its covalent attachment to residues K160/K170 of *Sa*-DHQ1 and *St*-DHQ1, respectively. From the model obtained by molecular replacement, and before inclusion of the inhibitor molecule, refinement was performed to obtain unbiased density for the inhibitor molecule and other model changes. A maximum-likelihood weighted *2Fo − Fc* map contoured at 1σ is shown up to 1.6 Å around the inhibitor molecule (yellow) and the catalytic lysine residue (green). The final model, including the inhibitor molecule, is superimposed onto the map. The distances are in angstroms.

Unbiased, calculated electron-density maps showed clear electron density for the ligand covalently bonded to the catalytic lysine residue (K160/K170 in *Sa*-DHQ1 and *St*-DHQ1, respectively) *via* an amine, which is visible in the two chains (Figure 3A). Remarkably, among all the lysine residues present in the two enzymes (13 for *Sa*-DHQ1 and 11 for *St*-DHQ1), only the catalytic lysine residue is modified by the ligand. Surprisingly, no electron density for the tertiary hydroxyl group in position C1 was identified in the two enzyme adducts (Figures 3B, C). Dehydration involving the C1–C2 bond took place to give a shikimate-like core, perhaps after covalent linkage at the C3 position in **6** *via* a similar mechanism to that observed for the natural substrate. Comparison of the structure of *St*-DHQ1 covalently modified by hydroxylamine **6** solved herein with the previously reported structure for the trapped enzyme-product Schiff base intermediate obtained by reduction with sodium borohydride (PDB ID 1QFE, 2.10 Å) (Gourley et al., 1999) showed that both structures are similar (rms = 0.3 between chains) (Supplementary Figure S2). Importantly, no cleavage of the amine bond promoted by the ligand was observed. Thus, compound **6** provides non-labile enzyme adducts by avoiding the intrinsic Schiff base hydrolysis performed by the enzyme.

In addition to the linkage to the catalytic lysine residue that blocks the enzyme active site, the enzyme-modified ligand is anchored to the enzyme *via* strong interactions with several conserved residues within the pocket. The latter comprises two main moieties in its chemical structure, which are similar for both structures (Figures 3B, C). Specifically: i) the carboxylate group in C1 establishes a salt-bridge with the guanidinium group of R202/R213, which has proven to be a key residue for recognition, along with a hydrogen bond with the amide side chain of Q225/Q236 (in *Sa*-DHQ1 and *St*-DHQ1, respectively), which is located on the substrate-covering loop that closes the active site for catalysis (Maneiro et al., 2014); and ii) the C4 and C5 hydroxyl groups establish a bidentate hydrogen bond with the carboxylate group of E35/E46, as well as hydrogen-bonding interactions with the guanidinium groups of R37/R48 and R70/R82 and with the carboxylate group of D102/D114 (in *Sa*-DHQ1 and *St*-DHQ1, respectively) *via* the structurally conserved water molecule, which is observed in all the reported crystal structures (such as PDB IDs 1SFJ (Nichols et al., 2004) and 1L9W (Lee et al., 2002)). Moreover, for both structures: i) a water molecule interacting with one of the oxygen atoms of the carboxylate group in the modified ligand *via* hydrogen bonding is observed [namely WAT100 (*Sa*-DHQ1) and WAT142 (*St*-DHQ1)]; and ii) the side chain of the catalytic histidine residue is located above the amino group of the modified lysine residue. For the *St*-DHQ1/**6** enzyme adduct, a long hydrogen bond between the C5 hydroxyl group and the side chain of residue S21, which is located on the flexible substrate-covering loop, is also identified. As a result, this loop folds more above the modified ligand. Finally, the active site of both adducts is closed by the substrate-covering loop, thus protecting the catalytic pocket from the water environment.

#### 2.4.2 *St*-DHQ1/8 enzyme complex

**FIGURE 4:**
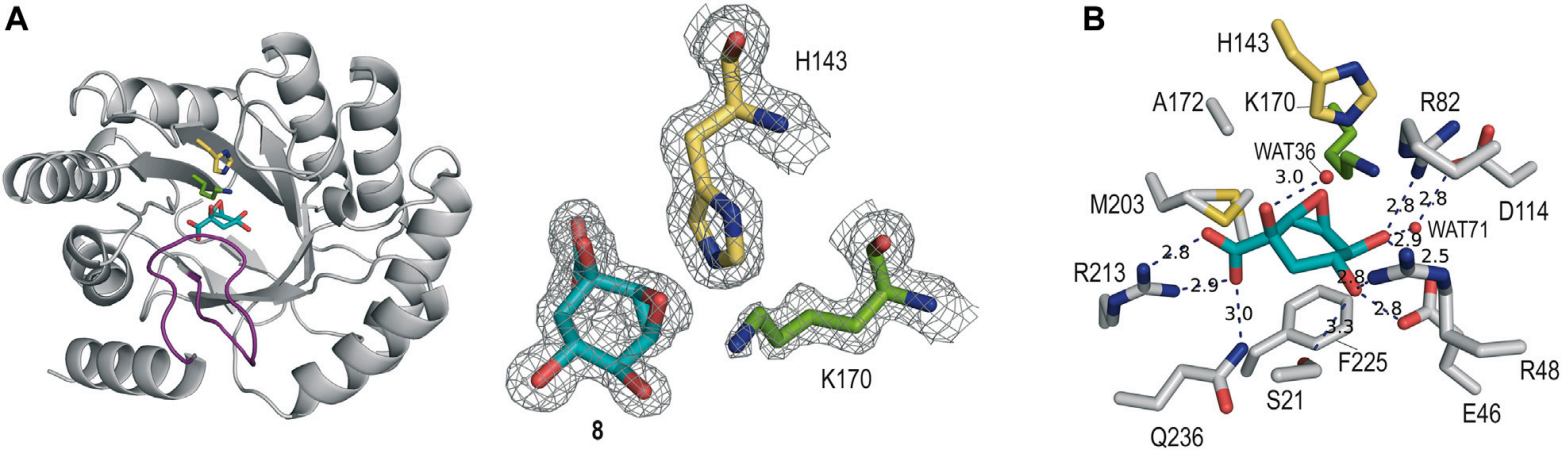
**(A)** Crystal structure of *St*-DHQ1 in complex with epoxide **8** (PDB ID 8B2C, 1.55 Å). Unbiased electron density for inhibitor **8** and the catalytic residues K170 and H143 of *St*-DHQ1. The substrate-covering loop is highlighted in purple. From the model obtained by molecular replacement and before inclusion of the inhibitor molecule, refinement was performed to obtain unbiased density for the inhibitor molecule and other model changes. A maximum-likelihood weighted 2Fo − Fc map contoured at 1σ is shown up to 1.6 Å around the inhibitor molecule (cyan) and residues K170 (green) and H143 (yellow). **(B)** Main contacts of epoxide **8** with *St*-DHQ1. Hydrogen-bonding and electrostatic interactions are shown as blue dashed lines. The distances are shown in angstrom. Only relevant residues are shown and labeled. Note how residue K170 shows a bent conformation.

The crystal structure of *St*-DHQ1 in complex with epoxide **8** crystallized with one molecule in the asymmetric unit and was solved at a resolution of 1.55 Å. The calculated maps revealed clear high electron density for the ligand molecule **8**, which binds to the active site *via* a salt bridge between its C1 carboxylate group and the guanidinium group of the conserved R213, along with a network of attractive hydrogen-bonding interactions with several side-chain residues (Figure 4A). Thus, as for adduct *St*-DHQ1/**6**, the ligand establishes direct contacts with the residues S21, E46, R48, R82, R213, and Q236, as well as two water molecules (WAT36 and WAT71; Figure 4B).

The enzyme structure shows that the introduction of a three-membered ring at the C2 and C3 positions of the cyclohexane ring in the ligand causes a twist in the overall chair conformation and, as a result, the strength of the contacts of the C5 hydroxyl group, as well as the accessibility to the C3 carbon by the ɛ-amino group of K170, are altered. Thus, the hydrogen bond between the C5 hydroxyl group in **8** and the carboxylate group of E46 is 0.3 Å longer than in the *St*-DHQ1/**6** adduct, while the interaction with residue R48 is shortened by 0.2 Å (Figures 4C vs 3C). More importantly, the CH group in the C3 position in **8** is twisted towards the bottom of the active site, thus hindering the reaction with the ɛ-amino group of K170, and the side chain of H143 is positioned close enough to the oxirane moiety in **8** (3.0 Å) for potential activation (Lewis acid). In addition, residue K170 shows a bent arrangement, and its ɛ-amino group is located pointing towards the carboxylate group of D114 (3.1 Å) and the structural water molecule (3.1 Å). The unsuitable orientation of the oxirane functional group within the cyclic compound might justify the lack of covalent modification observed experimentally.

#### 2.4.3 MALDI studies

The covalent modification of DHQ1 by compound **6** was also corroborated by mass spectrometry analysis of samples incubated with and without ligand. The MALDI spectra of *St*-DHQ1 enzyme incubated with **6** in ∼1:120 enzyme/ligand ratio for 24 h and at 25°C showed a peak for a covalently modified enzyme corresponding to an additional mass of 158 (theoretical value of the increased mass = 156; Supplementary Figure S3). No modifications were identified in similar studies carried out with compounds **7** and **8**.

#### 2.4.4 Molecular dynamics simulation studies

Of the two aminoquinic acid derivatives reported herein (compounds **6** and **7**), only the former, which has a hydroxyamino group, causes covalent modification of the catalytic lysine residue. However, from a structural point of view, the two compounds have a nitrogen moiety with an *R* configuration in the C3 position, and the overall geometry for recognition appears to be similar. Intrigued by this finding, the molecular basis of these differences was explored by computational studies. To that end, models of the corresponding enzyme complexes (Michaelis) for subsequent covalent modification were generated, and the stability of the ligands, and interactions with the residues of the active site, were analyzed by performing MD simulation studies. The required DHQ1/**6** and DHQ1/**7** binary complexes were constructed using the enzyme geometries found in the structures reported herein, after manual replacement of the observed ligand by compounds **6** and **7**. The resulting binary complexes were inmersed in a truncated octahedron box of water molecules and then subjected to 100 ns of dynamic simulation using the molecular mechanics force field AMBER ff14SB and GAFF (Wang et al., 2004).

Considering that the DHQ1 active site comprises various positively charged residues in the vicinity of the reactive pocket (Lys/His), specifically R70/R82 and R37/R48 (*Sa*-DHQ1/*St*-DHQ1, respectively), a key point in the recognition of compounds **6** and **7** appears to be the protonation state of its nitrogen substituent, as well as that for the catalytic histidine residue. For mechanistic considerations, the catalytic lysine residue was considered to be neutral. Thus, simulation studies with the two plausible protonation states (neutral and protonated) for the NH_2_ substituent in **7** and NHOH group in **6**, along with the catalytic histidine residue [neutral (δ protonation) and dual (δ and ε protonation)], were carried out, and the stability of the ligand and its contacts within the pocket were analyzed during the whole simulation, as discussed below.

**FIGURE 5:**
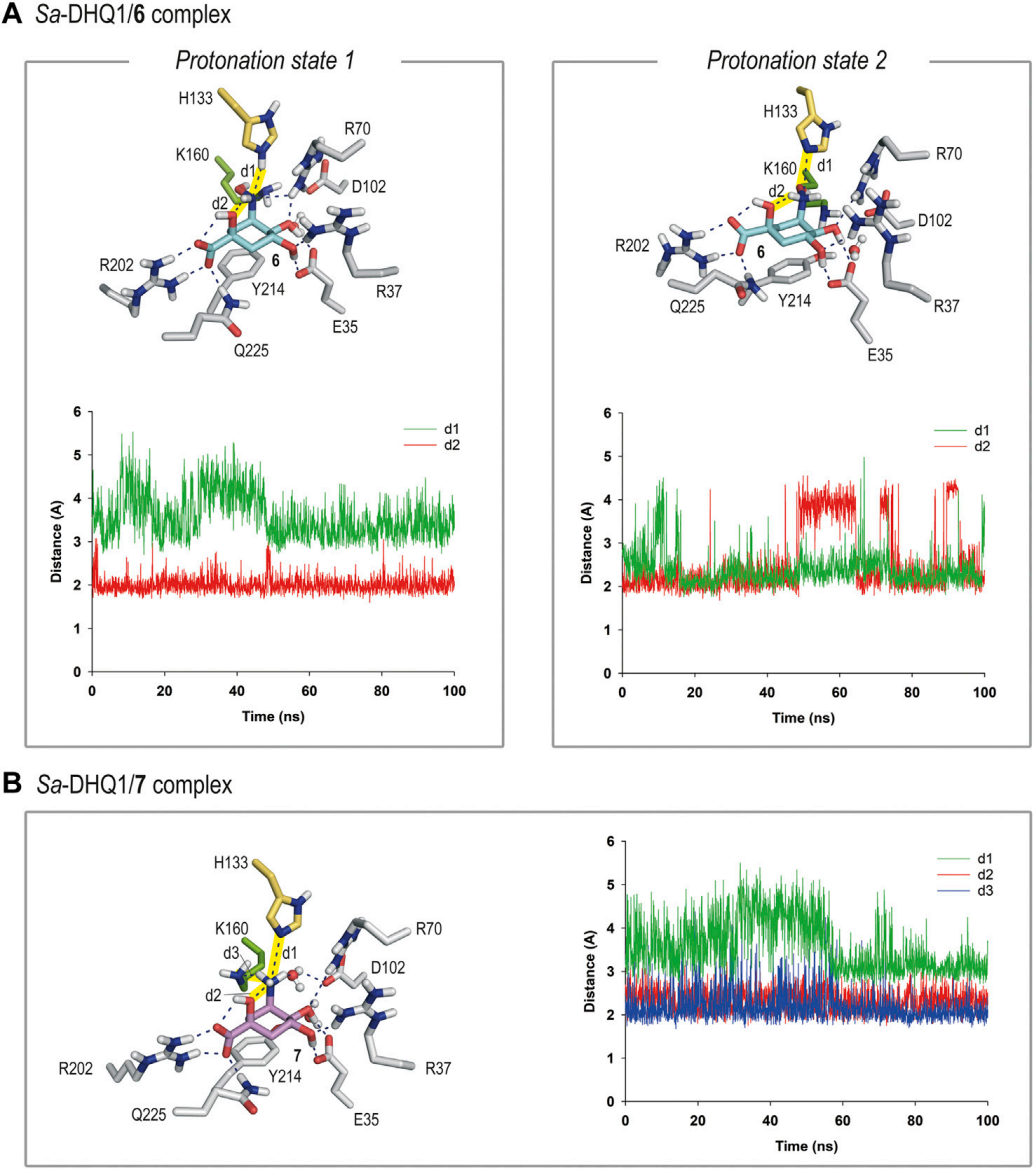
**(A)** Binding mode of compound **6** in the active site of *Sa*-DHQ1, as obtained by MD simulation studies, in which the two plausible protonation states of the NHOH moiety in **6** and residue H133 were considered. Snapshots taken after 80 (left) and 100 ns (right) are provided. Hydrogen-bonding and electrostatic interactions are shown as blue dashed lines. Only relevant residues are shown and labeled. Variation of the distances between H133 (HE2 or NE2 atoms) and the nitrogen atom in **6** (d1) and the NH group in **6** and its C1 hydroxyl group (oxygen atom, d2) in the *Sa*-DHQ1/**6** binary complex during the whole simulation considering protonation states 1 and 2. The distances studied are highlighted with yellow shading in the protein figure. **(B)** Binding mode of compound **7** (ammonium salt) in the active site of *Sa*-DHQ1 obtained by MD simulation studies. Residue H133 was considered in its neutral form. Snapshot taken after 100 ns of simulation. Variation of the distances between the ammonium group in **7** (H or N atoms) and H133 (NE2 atom, d1), its tertiary hydroxyl group (oxygen atom, d2) and K170 (NZ atom, d3) in the *Sa*-DHQ1/**7** binary complex during the whole simulation. The distances studied are highlighted with yellow shading in the protein figure. Note how the position of the catalytic lysine residue is frozen close to the C2 pocket by a strong hydrogen bond with the ammonium group in **7**, with an average distance of 2.2 Å during the simulation.

For compound **6**, our simulation studies revealed that the ligand would be stable in the active site when either: 1) its NHOH substituent is in the neutral form and the catalytic histidine residue is protonated; or 2) the catalytic histidine residue is neutral and the ligand is protonated on its nitrogen atom, as no significant changes were observed during the whole simulation (Figures 5A and Supplementary Figure S4). Thus, analysis of the root-mean-square deviation (rmsd) plots for the protein backbone and the ligand calculated from the MD simulation of the complexes revealed low values (Supplementary Figure S5).

In both cases, the arrangement of the NHOH substituent would be frozen by a defined network of hydrogen bonds. Thus, for option 1, the orientation of the NHOH group would be locked by three hydrogen bonds: one between the oxygen lone pair of the C1 hydroxyl group and the C3 aminic proton in the ligand, and two contacts involving the nitrogen lone pair in **6**, one with the NH group ε) of the protonated histidine residue and other with one of the NH moieties of the guanidinium group of residue R70. These interactions were found to be very stable, as revealed by an analysis of the variation of the distances between the atoms involved during the simulation (Figure 5A). In addition, the lysine side-chain was allocated to the reactive pocket close to the C3 carbon atom in **6**. As the estimated p*K*_a_ values for hydroxylamine and a histidine residue are rather similar (about 6), it seems reasonable that both protonation states might exist (Boulet et al., 1999). On the other hand, the superposition of the reported crystal structures of the DHQ1/**6** enzyme adducts with those obtained by MD simulations, in which the two plausible protonation states of the catalytic residues were considered, suggested that the modified lysine residue is likely to be protonated whereas the histidine residue is probably neutral (Supplementary Figure S6). Thus, other protonation scenarios would cause a significant motion in residues H133/H143 and D102/D114 (in *Sa*-DHQ1 and *St*-DHQ1, respectively) as well as the structural water molecule, all of which participate in stabilization of the adduct. On the other hand, the basicity of the amino substituent in **6** seems to be relevant for achieving lysine-covalent modification, since (–)-quinic acid (**9**), which possesses a hydroxyl group in the same position, does not form a covalent adduct with the enzyme, as revealed by the crystal structure of the *Salmonella enterica* DHQ1 enzyme in complex with **9** (PDB ID 4GUI, 1.78 Å) (Light et al., 2014).

In contrast, the results of the studies carried out with the DHQ1/**7** binary complex revealed that only the ammonium salt of **7** would be stable in the active site, in agreement with the estimated p*K*_a_ value for an amine (>9) (Figure 5B). More importantly, the ammonium group in **7** appears to freeze the position of the ε-amino group of K160 close to the C2 pocket by way of a strong hydrogen bond with the ammonium group in **7**, with an average distance between the atoms involved of 2.2 Å during the whole simulation (Figure 5B). Similar outcomes were obtained with the *S. aureus* and *S. typhi* enzymes (Supplementary Figures S7, S8). As for compound **6**, the ammonium substituent also establishes an internal hydrogen bond with the C1 hydroxyl group and an electrostatic interaction with H133. When the neutral form of the amino group was considered, large motion in the ligand, along with opening of the substrate-covering loop, was observed. To our knowledge, this is the first example of a secondary hydroxylamine derivative that cause the selective covalent modification of a sterically hindered lysine residue of an enzyme. Our outcomes support the potential of the hydroxyamino moiety as latent electrophile for lysine-targeted irreversible inhibition, when an exquisite anchoring of the ligand to the enzyme is achieved. The latter entails to study in-deep the enzyme recognition pattern, and its implementation into the scaffold to accomplish the required geometric perfection for reaction. More work must be done to improve inhibitor permeability into cells, for which aim an ester prodrug approch seems suitable as we demostrated before for inhibitors targeting the enzyme type II dehydroquinase (Tizón et al., 2011).

## 3 Conclusion

The results of the present study show that replacement of the C3 hydroxy group of (–)-quinic acid by a hydroxyamino substituent (compound **6**) results in a time-dependent irreversible inhibitor, whereas compound **7**, in which the latter functionality is replaced by an amino group, does not allow covalent modification of the enzyme. This finding was supported by resolution of the crystal structures for the DHQ1 from *Staphylococcus aureus* and *Salmonella typhi*, covalently modified by **6**, at 1.65 and 1.90 Å, respectively, which revealed that the inhibitor selectively reacts with the catalytic lysine residue to form an amine. The modified ligand also undergoes dehydration at the tertiary hydroxyl group to afford a shikimate-like derivative. This structure is similar to that reported previously for the trapped enzyme-product Schiff-base intermediate obtained by reduction with sodium borohydride. Despite having a reactive electrophilic warhead, compound **8** proved to be a reversible competitive inhibitor of the enzyme and was very stable in the enzyme active site, as evidenced by resolution of the crystal structure of the *Salmonella typhi* DHQ1 enzyme in complex with **8** at 1.55 Å. The twisted arrangement of the cyclohexane ring in **8** towards residue F255 observed in the crystal structure, which is located on the bottom part of the active site, seems to hinder access of the essential lysine residue for nucleophilic attack.

The structures of the enzyme DHQ1 reported herein, in combination with MD simulation studies, provide valuable information relating to the binding modes of the targeted compounds, as well as insights into the molecular basis by which the quinate-based compounds explored are activated for lysine-covalent modification or not. Our simulation studies suggest that compound **7**, the amino group of which would be protonated at the active site, could prevent covalent modification of the catalytic lysine residue by freezing the position of the enzyme nucleophile (ɛ-amino Lys group) on the same face as the leaving group (Re face). The catalytic histidine residue, which would be in its neutral form to avoid electrostatic repulsion with the ammonium group in **7**, would also be anchored in the vicinity of the substituent by a strong hydrogen-bonding interaction. In contrast, the latter unreactive arrangement would be avoided, to some extent, by replacement of the amino group in **7** by the NHOH moiety, since in the latter case a neutral form of the leaving group in the ligand would be achieved upon binding, thus facilitating the required flexibility of the lysine residue for reaction. In addition, the neutral state of the NHOH substituent in **6** would allow the dual protonation state of the catalytic histidine residue, and the establishment of a strong hydrogen-bonding interaction between both moieties. Although additional computational studies are necessary to validate this hypothesis, the arrangement identified in the present study for the DHQ1/**6** binary complex suggests a covalent modification mechanism consisting of nucleophilic attack of the lysine residue at C3 in **6** with release of NH_2_OH mediated by the essential histidine residue acting as a proton donor.

## 4 Experimental

### 4.1 General

All starting materials and reagents were commercially available and were used without further purification. ^1^H (300 and 500 MHz) and ^13^C NMR spectra (75 and 125 MHz) were measured in deuterated solvents. *J* values are given in Hertz. NMR assignments were carried out by a combination of 1D, COSY, and DEPT-135 experiments. FTIR spectra were recorded in a PerkinElmer Two FTIR spectrometer with attenuated total reflection. Melting points were measured in a Büchi M-560 apparatus. ${\left[\alpha \right]}_{D}^{20}$ values are given in 10^–1^ deg cm^2^ g^−1^. Milli-Q deionized water was used in all buffers. The spectroscopic measurements were performed using a Varian Cary 100 UV-Vis spectrophotometer with a 1 cm path-length cell fitted with a Peltier temperature controller. Protein analysis was performed using a MALDI TOF/TOF Mass Spectrometer (4,800 Analyzer, AbSciex). The ProteoMass™ MALDI calibration kit (Merck) was employed for calibration and sinapic acid as a matrix. Protein spectra were analyzed using the Data Explorer™ Software.

### 4.2 Methyl (1*R*,3*S*,4*S*,6*R,9S*)-7-hydroxyimino-9-hydroxy-3,4-dimethoxy-3,4-dimethyl-2,5-dioxabicyclo[4.4.0]decane-9-carboxylate (11)

A suspension of ketone **10** (305 mg, 0.96 mmol) was prepared in two steps from commercially available (–)-quinic acid **9**), as described previously (Toscano et al., 2003). Thus, a mixture of hydroxylamine hydrochloride (100 mg, 1.44 mmol) and sodium acetate trihydrate (196 mg, 1.44 mmol) in acetonitrile (8 mL) and water (3.2 mL) was stirred at room temperature for 24 h. The solvent was then removed under reduced pressure and the resulting residue was dissolved in dichloromethane and water. The organic layer was separated, dried (anh. Na_2_SO_4_), filtered and concentrated under reduced pressure. The resulting mixture was then purified by flash chromatography, eluting with diethyl ether, to yield compound **11** (286 mg, 89%) as a white foam. ${\left[\alpha \right]}_{D}^{20}$ = +87.4° (c1.0, CHCl_3_). ^1^H NMR (300 MHz, CDCl_3_) δ: 8.60 (s, 1H, OH), 4.29 (d, *J* = 9.9 Hz, 1H, H6), 4.18–4.09 (m, 1H, H1), 3.81 (s, 3H, OCH_3_), 3.53 (dd, *J* = 2.7 and 14.6 Hz, 1H, H8_eq_), 3.27 (s, 3H, OCH_3_), 3.22 (s, 3H, OCH_3_), 3.21 (br s, 1H, OH), 2.19 (d, *J* = 15.3 Hz, 1H, H8_ax_), 2.09 (d, *J* = 11.6 Hz, 1H, H10_ax_), 2.00 (ddd, *J* = 2.7, 4.7 and 13.1 Hz, 1H, H10_eq_), 1.36 (s, 3H, CH_3_) and 1.29 (s, 3H, CH_3_) ppm. ^13^C NMR (63 MHz, CDCl_3_) δ: 174.8 C), 149.9 C), 100.1 C), 99.7 C), 74.0 C), 71.8 (CH), 67.5 (CH), 53.2 (OCH_3_), 48.2 (OCH_3_), 47.8 (OCH_3_), 37.4 (CH_2_), 33.3 (CH_2_), 17.7 (CH_3_) and 17.6 (CH_3_) ppm. FTIR (ATR): 3305 (OH) and 1734 (CO) cm^−1^. MS (ESI) *m/z* = 334 (MH^+^). HRMS calcd for C_14_H_24_NO_8_ (MH^+^): 334.1496; found, 334.1496.

### 4.3 Methyl (1*R*,3*S*,4*S*,6*R,7R,9S*)-7-(*O*-benzylhydroxyl)amino-3,4-dimethoxy-3,4-dimethyl-2,5-dioxabicyclo[4.4.0]decane-9-carboxylate (12)

A suspension of ketone **10** (500 mg, 1.57 mmol), *O*-benzylhydroxylamine hydrochloride (250.6 mg, 1.57 mmol), anhydrous sodium acetate (258 mg, 3.14 mmol) and 4 Å molecular sieves (500 mg) in dry methanol (8.0 mL) was stirred for 20 h at room temperature and under an inert atmosphere. The reaction mixture was filtered through a plug of Celite^®^ and washed with methanol. The filtrate and washings were concentrated under reduced pressure. The resulting residue was dissolved in glacial acetic acid (3.9 mL), at room temperature and under an inert atmosphere, and then treated with sodium cyanoborohydride (197 mg, 3.14 mmol). After stirring for 1 h, the solvent was removed under reduced pressure and the resulting residue was dissolved in ethyl acetate and saturated NaHCO_3_. The aqueous layer was separated, dried (anh. Na_2_SO_4_), filtered and concentrated under reduced pressure. The reaction mixture was purified by flash chromatography, eluting with (50:50) diethyl ether/hexane, to yield compound **12** (489 mg, 73%) as a white foam. ${\left[\alpha \right]}_{D}^{20}$ = +104.9 (*c*1.0, CHCl_3_). ^1^H NMR (300 MHz, CDCl_3_) δ: 7.38–7.34 (m, 5H, 5×ArH), 4.93 (d, *J* = 10.8 Hz, 1H, OC*H*H), 4.82 (d, *J* = 10.8 Hz, 1H, OCH*H*), 4.04 (dt, *J* = 4.9 and 10.7 Hz, 1H, H1), 3.78 (s, 3H, OCH_3_), 3.76–3.73 (m, 1H, H6), 3.62–3.59 (m, 1H, H7), 3.24 (s, 3H, OCH_3_), 3.22 (s, 3H, OCH_3_), 2.48 (td, *J* = 2.7 and 14.9 Hz, 1H, C*H*H), 2.18–2.11 (m, 1H, C*H*H), 1.92 (dd, *J* = 3.0 and 14.9 Hz, 1H, CH*H*), 1.87 (t, *J* = 12.4 Hz, 1H, CH*H*), 1.28 (s, 3H, CH_3_) and 1.27 (s, 3H, CH_3_) ppm. ^13^C NMR (75 MHz, CDCl_3_) δ: 173.2 C), 136.2 C), 128.2 (2×CH), 128.1 (2×CH), 127.9 (CH), 100.0 C), 99.4 C), 76.3 C), 75.6 (CH_2_), 70.4 (CH), 62.9 (CH), 58.6 (CH), 52.2 (OCH_3_), 47.6 (OCH_3_), 47.6 (OCH_3_), 38.6 (CH_2_), 33.1 (CH_2_), 17.5 (CH_3_) and 17.3 (CH_3_) ppm. FTIR (ATR): 3317 (OH + NH) and 1737 (CO) cm^−1^. MS (ESI) *m/z* = 425 (MH^+^). HRMS calcd for C_21_H_32_NO_8_ (MH^+^): 426.2122; found, 426.2136.

### 4.4 Methyl (1*R*,3*S*,4*S*,6*R,7R,9S*)-7-(*tert*-butoxycarbonyl)amino-3,4-dimethoxy-3,4-dimethyl-2,5-dioxabicyclo[4.4.0]decane-9-carboxylate (13)

A suspension of Pd/C (18 mg, 10%) in methanol (2.5 mL) and 5 drops of acetic acid was treated, under a hydrogen atmosphere and at room temperature, with a solution of the *O*-benzylhydroxylamine **12** (181 mg, 0.43 mmol) in methanol (2.5 mL) via *canula*. The resulting suspension was stirred at room temperature for 18 h. After removal of the hydrogen atmosphere, the suspension was filtered through a plug of Celite^®^. The filtrate and washings (methanol) were concentrated under reduced pressure. A solution of the resulting residue in dry DMF (4.5 mL) was treated successively with dry triethylamine (0.1 mL, 0.65 mmol) and di-*tert*-butyldicarbonate (113.5 mg, 0.52 mmol) and stirred for 3 h. The reaction mixture was diluted with ethyl acetate and water. The organic layer was separated, and the aqueous layer was extracted with ethyl acetate (×2). The combined organic extracts were dried (anh. Na_2_SO_4_), filtered and concentrated under reduced pressure. The residue was purified by flash chromatography, eluting with (80:20) diethyl ether/hexane, to yield compound **13** (85.5 mg, 47%) as a white foam. ${\left[\alpha \right]}_{D}^{20}$ = +116.5 (*c*1.0, CHCl_3_). ^1^H NMR (300 MHz, CDCl_3_) δ: 5.54 (d, *J* = 9.0 Hz, 1H, NH), 4.16 (m, 1H, H7), 4.06–3.97 (m, 1H, H1), 3.75 (s, 3H, OCH_3_), 3.58 (dd, *J* = 4.5 and 10.3 Hz, 1H, H6), 3.22 (s, 3H, OCH_3_), 3.21 (s, 3H, OCH_3_), 2.00 (dd, *J* = 4.3 and 14.5 Hz, 1H, C*H*H), 1.94–1.87 (m, 3H, CH*H* + CH_2_), 1.41 (s, 9H, 3×CH_3_) and 1.23 (s, 6H, 2×CH_3_) ppm. ^13^C NMR (75 MHz, CDCl_3_) δ: 175.7 C), 156.0 C), 100.0 C), 99.6 C), 79.1 C), 75.3 C), 71.5 (CH), 62.9 (CH), 53.3 (OCH_3_), 53.3 (OCH_3_), 48.3 (CH), 48.0 (2×OCH_3_), 38.4 (CH_2_), 37.1 (CH_2_), 28.5 (3×CH_3_), 17.9 (CH_3_) and 17.7 (CH_3_) ppm. FTIR (ATR): 3347 (OH + NH) 1725 (CO) and 1,694 (CO) cm^−1^. MS (ESI) *m/z* = 420 (MH^+^). HRMS calcd for C_19_H_34_NO_9_ (MH^+^): 420.2228; found, 420.2223. The configuration of the C7 position was determined by NOE experiments. Thus, irradiation of the H7 signal (4.16 ppm) led to enhancement of the H6 (3.58 ppm) and NH (5.54 ppm) signals by 5.2% and 1.3%, respectively.

### 4.5 Methyl (1*R*,3*S*,4*S*,6*R,7R,9S*)-7-hydroxylamino-3,4-dimethoxy-3,4-dimethyl-2,5-dioxabicyclo[4.4.0]decane-9-carboxylate (14)

NaBH_3_CN (27 mg, 0.42 mmol) and HCl (0.22 mL, 2.0 M) were added to a stirred solution of compound **11** (93 mg, 0.28 mmol) in methanol (0.45 mL) at room temperature and under an inert atmosphere. After stirring at room temperature for 3 h, powdered Na_2_CO_3_ (anh.) was added and the solvent was concentrated under reduced pressure. The reaction mixture was partitioned with ethyl acetate and water, the aqueous layer was separated, and the organic layer was washed with Na_2_CO_3_ (sat), dried (anh. Na_2_SO_4_), filtered, and concentrated under reduced pressure. The resulting residue was purified by flash chromatography on silica gel, eluting with (90:10) diethyl ether/hexane, to afford compound **14** (80 mg, 85%) as a white foam. ${\left[\alpha \right]}_{D}^{20}$ = +88.9 (*c*1.0, CHCl_3_). ^1^H NMR (300 MHz, CDCl_3_) δ: 6.56 (s, 1H, NH), 4.12–4.03 (m, 1H, H1), 3.81–3.75 (m, 1H, H6), 3.74 (s, 3H, OCH_3_), 3.52 (m, 1H, H7), 3.23 (s, 3H, OCH_3_), 3.22 (s, 3H, OCH_3_), 2.42 (d, *J* = 14.6 Hz, 1H, CH*H*-8), 2.06 (m, 1H, CH*H*-10), 1.93 (t, *J* = 12.4 Hz, 1H, C*H*H-10), 1.78 (dd, *J* = 14.5 and 2.5 Hz, 1H, C*H*H-8) and 1.26 (s, 6H, 2×CH_3_) ppm. ^13^C NMR (63 MHz, CDCl_3_) δ: 173.9 C), 100.4 C), 99.8 C), 76.5 C), 70.9 (CH), 63.3 (CH), 60.1 (CH), 52.7 (OCH_3_), 48.1 (2×OCH_3_), 38.6 (CH_2_), 33.0 (CH_2_), 17.9 (CH_3_) and 17.7 (CH_3_) ppm. FTIR (ATR): 3427 (OH), 3385 (NH) and 1751 (CO) cm^−1^. MS (ESI) *m/z* = 358 (MNa^+^). HRMS calcd for C_14_H_25_NO_8_Na (MNa^+^): 358.1472; found, 358.1476. The configuration of the C7 position was determined by NOE experiments. Thus, irradiation of the H8_ax_ signal (1.78 ppm) led to enhancement of the H6 (3.81–3.75 ppm) and H7 (3.52 ppm) signals by 6.2% and 4.2%, respectively.

### 4.6 3-Hydroxyaminoquinic acid [(1*S*,3*R*,4*R*,5*R*)-1,3,4-trihydroxy-5-hydroxylaminocyclohexane-1-carboxylic acid], hydrochloride form (6)

A solution of compound **14** (71 mg, 0.21 mmol) in HCl (2.1 mL, 0.3 M) was heated at 100°C for 24 h. After cooling to room temperature, the solution was washed with ethyl acetate (×3). The aqueous layer was lyophilized to give compound **6** (41 mg, 94%) as a brown foam. ${\left[\alpha \right]}_{D}^{20}$ = −18.8 (*c*1.0, H_2_O). ^1^H NMR (300 MHz, D_2_O) δ: 4.0 (m, 2H, H5+H4), 3.93 (m, 1H, H3), 2.25 (m, 2H, CH_2_) and 2.17–2.02 (m, 2H, CH_2_). ^13^C NMR (63 MHz, D_2_O) δ: 179.1 C), 76.0 C), 71.2 (CH), 69.3 (CH), 63.6 (CH), 40.7 (CH_2_) and 31.7 (CH_2_) ppm. FTIR (ATR): 3088 (OH), 2,925 (NH) and 1715 (CO) cm^−1^. MS (ESI) *m/z* = 206 (M−H). HRMS calcd for C_7_H_12_NO_6_ (M−H): 206.0670; found, 206.0671.

### 4.7 3-Aminoquinic acid [(1*S*,3*R*,4*R*,5*R*)-3-amino-1,4,5-trihydroxycyclohexane-1-carboxylic acid], hydrochloride form (7)

A solution of compound **13** (83.5 mg, 0.2 mmol) in an aqueous solution of HCl (2 mL, 0.3 M) was heated at 100 °C for 24 h. After cooling to room temperature, the solution was washed with ethyl acetate (×3). The aqueous layer was lyophilized to give compound **7** (45.5 mg, 90%) as a white foam. ${\left[\alpha \right]}_{D}^{20}$ = −19.2 (*c*1.0, H_2_O). ^1^H NMR (300 MHz, D_2_O) δ: 4.02–3.94 (m, 1H, H5), 3.85–3.78 (m, 2H, H3+H4), 2.28 (dd, *J* = 4.3 and 15.3 Hz, 1H, C*H*H), 2.20–2.11 (m, 2H, C*H*H + CH*H*) and 1.96 (dd, *J* = 13.6 and 10.4 Hz, 1H, CH*H*), ppm. ^13^C NMR (63 MHz, D_2_O) δ: 176.9 C), 74.4 C), 70.6 (CH), 66.1 (CH), 51.2 (CH), 39.2 (CH_2_) and 32.8 (CH_2_) ppm. FTIR (ATR): 3320 (NH), 3109 (OH) and 1739 (CO) cm^−1^. MS (ESI) *m/z* = 190 (M−H). HRMS calcd for C_7_H_12_NO_5_ (M−H): 190.0721; found, 190.0722.

### 4.8 (1*R*,2*S*,3*S*,4*S*,5*R*)-2,3-epoxi-1,4-dihydroxycyclohexan-1,5-carbolactone (19)

A solution of silyl ether **18** (53 mg, 0.17 mmol) in (20:1) TFA/H_2_O (1.7 mL) was stirred at room temperature for 5 days. The solvents were then evaporated under reduced pressure and the resulting residue was purified by flash chromatography on silica gel, eluting with diethyl ether, to give alcohol **19** (20 mg, 68%) as a colorless oil. ${\left[\alpha \right]}_{D}^{20}$ = −110.2° (*c*1.7, CH_3_OH). ^1^H NMR (300 MHz, acetone-d6) δ: 4.35 (td, *J* = 2.4 and 6.3 Hz, 1H, H5), 3.98 (m, 1H, H4), 3.43 (dd, *J* = 1.5 and 4.2 Hz, 1H, H2), 3.37 (td, *J* = 2.1 and 4.2 Hz, 1H, H3), 3.10 (br s, 2H, 2×OH), 2.61 (d, *J* = 11.7 Hz, 1H, CH*H*) and 2.09 (m, 1H, C*H*H) ppm. ^13^C NMR (63 MHz, acetone-*d*6) δ: 176.6 C), 78.2 (CH), 75.2 C), 66.2 (CH), 57.6 (CH), 53.0 (CH) and 33.3 (CH_2_) ppm. FTIR (ATR): 3397 (OH) and 1771 (CO) cm^−1^.

### 4.9 (1*S*,2*R*,4*R*,5*S*,6*S*)-2,4,5-trihydroxy-7-oxabicyclo[4.1.0]heptane-2-carboxylic acid (8)

A solution of lactone **19** (30 mg, 0.17 mmol) in THF (1.7 mL) was treated with aqueous LiOH (0.86 mL, 0.5 M) and the resulting mixture was stirred at room temperature for 30 min. The reaction mixture was diluted with Milli-Q water, and THF was evaporated under reduced pressure. The resulting aqueous solution was washed with diethyl ether (×3) and the aqueous extract was treated with Amberlite IR-120 (H^+^) until pH 6. The resin was filtered off and washed with milli-Q water. The filtrate and washings were lyophilized to give acid **8** (31 mg, 97%) as a white foam. ${\left[\alpha \right]}_{D}^{20}$ = −16.1° (*c*2.2, H_2_O). 1H NMR (250 MHz, D_2_O) δ: 3.83 (dd, *J* = 1.5 and 8.5 Hz, 1H, H5), 3.73 (m, 1H, H4), 3.52 (dd, *J* = 1.5 and 4.0 Hz, 1H, H5), 3.47 (d, *J* = 4.0 Hz, 1H, H1) and 1.84 (m, 2H, CH_2_−3) ppm. ^13^C NMR (63 MHz, D_2_O) δ: 177.9 C), 73.7 C), 72.8 (CH), 66.5 (CH), 59.1 (CH), 58.3 (CH) and 41.4 (CH_2_) ppm. FTIR (ATR): 3354 (OH) and 1722 (CO) cm^−1^. MS (ESI) *m/z* = 189 (M−H). HRMS calcd for C_7_H_9_O_6_ (M−H): 189.0405; found: 189.0413.

### 4.10 DHQ1 assays

The *St*-DHQ1 and *Sa*-DHQ1 enzyme were purified as described previously (Moore et al., 1993; Nichols et al., 2004). Concentrated solutions of *St*-DHQ1 (0.85 mg mL^−1^, 30.74 µM) and *Sa*-DHQ1 (4 mg mL^−1^, 148.13 µM) were stored in potassium phosphate buffer (PPB) (50 mM) and DTT (1 mM) at pH 6.6 and −80°C. When required for assays, aliquots of the enzyme stocks were diluted in water and buffer and stored on ice. DHQ1 was assayed in the forward direction by monitoring the increase in absorbance at 234 nm in the UV spectrum due to the absorbance for the enone-carboxylate chromophore of 3-dehydroshikimic acid (ε/M^−1^ cm^−1^ 12 000). Standard assay conditions were PPB (50 mM, pH 7.2) at 25°C. Each assay was initiated by addition of the substrate. Solutions of 3-dehydroquinic acid **1**) were calibrated by equilibration with DHQ1 and measurement of the change in the UV absorbance at 234 nm due to formation of the enone-carboxylate chromophore of 3-dehydroshikimic acid. Under assay conditions, the kinetic parameters were: i) *Sa*-DHQ1: *K*_*m*_ = 18 ± 3 μM; *k*_cat_ = 255 ± 12 s^−1^; ii) *St*-DHQ1: *K*_*m*_ = 24 ± 3 μM; *k*_cat_ = 1.1 ± 0.1 s^−1^. The program GraFit 7.0.3. (Erithacus Software Ltd.). was used for data fitting.

### 4.11 Inhibition assays

Incubation of *Sa-*DHQ1 and *St*-DHQ1 (2.96 µM from a stock protein concentration of 0.85 mg mL^−1^ and 4 mg mL^−1^, respectively) with various aqueous solutions of compounds **6**–**8** (30–4,000 µM) was carried out in PPB (0.5 mL, 50 mM) at pH 7.2 at 25°C. The activity was determined progressively in duplicate over a 3 h period under the standard assay conditions (see above) using aliquots from the incubation samples and the control. Semilogarithmic plots of residual activity as a function of time with a range of inhibitor concentrations [I] were performed to obtain the first-order rate constant, *k*_*obs,*_ calculated from the slope, and is related with *k*_*inact*_ K_I_ constants by Equation 1 implemented in GraFit (Plapp, 1982):

$$k_{obs}=(k_{\mathrm{inact}}\;\left[I\right])\;/\;\left(K_{I}\right)+\left[I\right])$$ (1)

IC_50_ values were determined in duplicate by measuring the initial rates at fixed enzyme and substrate concentrations (14 µM) in the absence and in the presence of various compounds concentrations, which was adjusted to the Equation 2 implemented in GraFit:

$${y=E}_{\max}\;/\;1+{({IC}_{50}\;/\;x)}^{n}$$ (2)

were y is the observed effect at inhibitor concentration x, E_max_ corresponds to the maximum effect, IC_50_ is the concentration at which a 50% growth inhibition is obtained, and n is the slope of the curve.

### 4.12 Incubation studies for mass spectrometry

*St*-DHQ1 (3.1 µM from a stock protein concentration of 0.85 mg mL^−1^) and *Sa-*DHQ1 (3.0 µM from a stock protein concentration of 4 mg mL^−1^) was incubated with various aqueous solutions of ligands **6**–**8** in PPB (0.5 mL, 50 mM) at pH 7.2, 25°C. The activity was determined progressively over a 24 h period under the standard assay conditions using aliquots from the incubation samples and the control. The activity was measured at a substrate concentration of 14 µM. The initial rate of each assay was measured. The samples were concentrated and washed (5 mM ammonium carbonate) for MALDI analysis by centrifugation at 4 °C using Amicon^®^ centrifugal filters (Amicon Ultra-10). The samples were freeze-dried, diluted with 5 mM ammonium bicarbonate (5 µL) and analyzed by mass spectrometry using a MALDI TOF/TOF Mass Spectrometer (4,800 Analyzer, AbSciex). The ProteoMass™ MALDI calibration kit (Merck) was employed for calibration and sanipic acid as a matrix. Spectra were analyzed using the Data Explorer™ Software. Experiments were performed in triplicate.

### 4.13 Crystallization of the DHQ1/6 adducts

*St*-DHQ1 (0.85 mg mL^−1^) and *Sa*-DHQ1 (4 mg mL^−1^) were concentrated to 11 and 20 mg mL^−1^, respectively, in 50 mM PPB pH 7.0, and 0.5 mM DDT. Ligands were dissolved at 250 mM in methanol and added to the protein solution at a ratio of 1:20 (v/v) to give a solution of approximately 10 equivalents of ligand per protein monomer. Crystals of up to: a) 0.2 mm × 0.2 mm of the *St*-DHQ1/**6** (plate-shape prims); and b) 0.4 mm × 0.01 mm of the *Sa*-DHQ1/**6** (needles) were obtained after 3 weeks of vapor diffusion in sitting drops comprised of 2.0 μL protein/ligand solution mixed with 2.0 μL reservoir solution against 0.15 mL reservoirs containing: i) for *St*-DHQ1: 12% PEG 2000 MME, 0.1 M MES pH 6.0; and ii) for *Sa*-DHQ1: 20% PEG 3350, 0.1 M Bis-Tris pH 5.5, 0.2 M Li_2_SO_4_.

### 4.14 Crystallization of the *St*-DHQ1/8 complex

Prism-shaped apo-*St*-DHQ1 crystals of up to 0.03 mm × 0.03 mm were obtained from solution of *St*-DHQ1 concentrated to 8 mg mL^−1^ in buffer A (10 mM Tris-HCl pH 7.4, 40 mM KCl) after 4 weeks of vapor diffusion in sitting drops comprised of 2.0 μL of protein solution mixed with 2.0 μL of reservoir solution and equilibrated against 0.15 mL reservoirs containing the crystallization mixture [26% PEG 2000 MME, 0.1 M Hepes pH 7.0]. *St*-DHQ1/**8** complex crystals were obtained after soaking of apo-*St*-DHQ1 crystals in 10 mM solutions of epoxide **8** in the crystallization mixture for 48 h.

### 4.15 Structure determination

Crystals were mounted into cryoloops and directly flash-frozen by rapid immersion in liquid nitrogen. For the *St*-DHQ1/**6** and *Sa*-DHQ1/**6** enzyme adducts, X-ray diffraction data were collected on beamline BL13-Xaloc (Juanhuix et al., 2014) (Alba Synchrotron, Barcelona, Spain, detector Pilatus 6M-Dectris) from a crystal maintained at 100 K. For the *St*-DHQ1/**8** enzyme complex, X-ray diffraction data were collected on beamline ID23–2 (ESRF; Grenoble, France) from a crystal maintained at 100 K. The diffraction data were processed, scaled, corrected for absorption effects and the crystal unit-cell parameters were calculated by global refinement with autoPROC (Vonrhein et al., 2011), which uses XDS (Kabsch 2010), AIMLESS (Evans 2006) and other programs from the CCP4 software suite (Winn et al., 2011).

The structures were solved by molecular replacement, using the program MOLREP (Vagin and Teplyakov 2010) with a search model generated from PDB entries 4CNN (*St*-DHQ1) and 6FSH (*Sa*-DHQ1), from which the ligand and solvent atoms were removed. The ligand structure and geometrical restraints, including the covalent link to the protein, were generated with the PRODRG2 (Schüttelkopf and van Aalten 2004) server and JLIGAND (Lebedev et al., 2012). The ligand was manually placed during model building, which was performed with COOT (Casañal et al., 2020). Reflections used to calculate Rfree (Brünger 1997) were selected randomly. Refinement of the models was performed with REFMAC5 (Kovalevskiy et al., 2018) and final structure validation was performed with MOLPROBITY (Williams et al., 2018) and the PDB validation server (Read et al., 2011). Data collection, refinement and model statistics are summarized in Table 1. Structure figures were prepared using PYMOL (DeLano 2002).

### 4.16 MD simulation studies

Minimization of the ligands, DHQ1/ligand adducts and binary complexes, and MD simulation of the resulting minimized adducts and complexes, were carried following our previously described protocol (Lence et al., 2020). The simulation time was 100 ns. The coordinates found in the crystal structures of *Sa*-DHQ1 and *St*-DHQ1 reported herein were employed. The stability and reliability of all the DHQ1/ligand adducts and complexes described herein were verified by analysing the root-mean-square deviation (rmsd) for the whole protein backbone (Cα, C, N and O atoms), the inhibitors and the modified ligands during the whole simulation by using the cpptraj module in AMBER 16.

## Data Availability

Coordinates and structure factors are available from the Protein Data Bank with accession codes 8B2A, 8B2B and 8B2C.
