# Supplementary material for: Quinate-based ligands for irreversible inactivation of the bacterial virulence factor DHQ1 enzyme—A molecular insight
Source: Front Mol Biosci. 2023 Jan 24;10:1111598. doi: 10.3389/fmolb.2023.1111598 (PMC9902378; doi:10.3389/fmolb.2023.1111598)
Supplement: Supplementary file 1 [file DataSheet1.PDF]

Supplementary Material

Quinate-Based Ligands for Irreversible Inactivation of the Bacterial Virulence Factor DHQ1 Enzyme – A Molecular Insight

Ángela Rodríguez[1], María Maneiro[1], Emilio Lence[1], José M. Otero[1], Mark J. van Raaij[2], Paul Thompson[3], Alastair R. Hawkins[3], Concepción González-Bello[1]*

[1]Centro Singular de Investigación en Química Biolóxica e Materiais Moleculares (CiQUS), Departamento de Química Orgánica, Universidade de Santiago de Compostela, Santiago de Compostela, Spain

[2]Departamento de Estructura de Macromoléculas, Centro Nacional de Biotecnología (CSIC), Madrid, Spain

[3]Newcastle University Biosciences Institute, The Medical School, Newcastle University, Newcastle upon Tyne, UK
* e-mail: concepcion.gonzalez.bello@usc.es

Table of Contents

1. Figure S1 .... S2
2. Figure S2 .... S3
3. Figure S3 .... S4
4. Figure S4 .... S5
5. Figure S5 .... S6
6. Figure S6 .... S7
7. Figure S7 .... S8
8. Figure S8 .... S9
9. Figure S9 .... S10
10. Figure S10 .... S11
11. Figure S11 .... S12

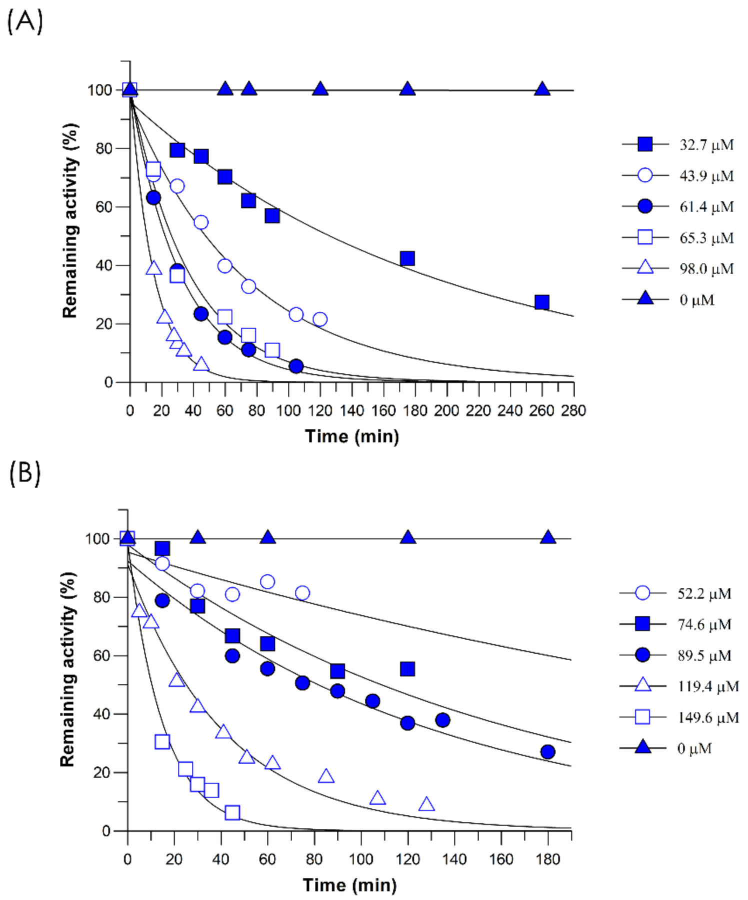

Figure S1. Inactivation of DHQ1 enzyme by incubation with compound **6**: (A) *Sa*-DHQ1; (B) *St*-DHQ1. Results are the average of three observations. Assay conditions were PPB (50 mM, pH 7.2) at 25 °C.

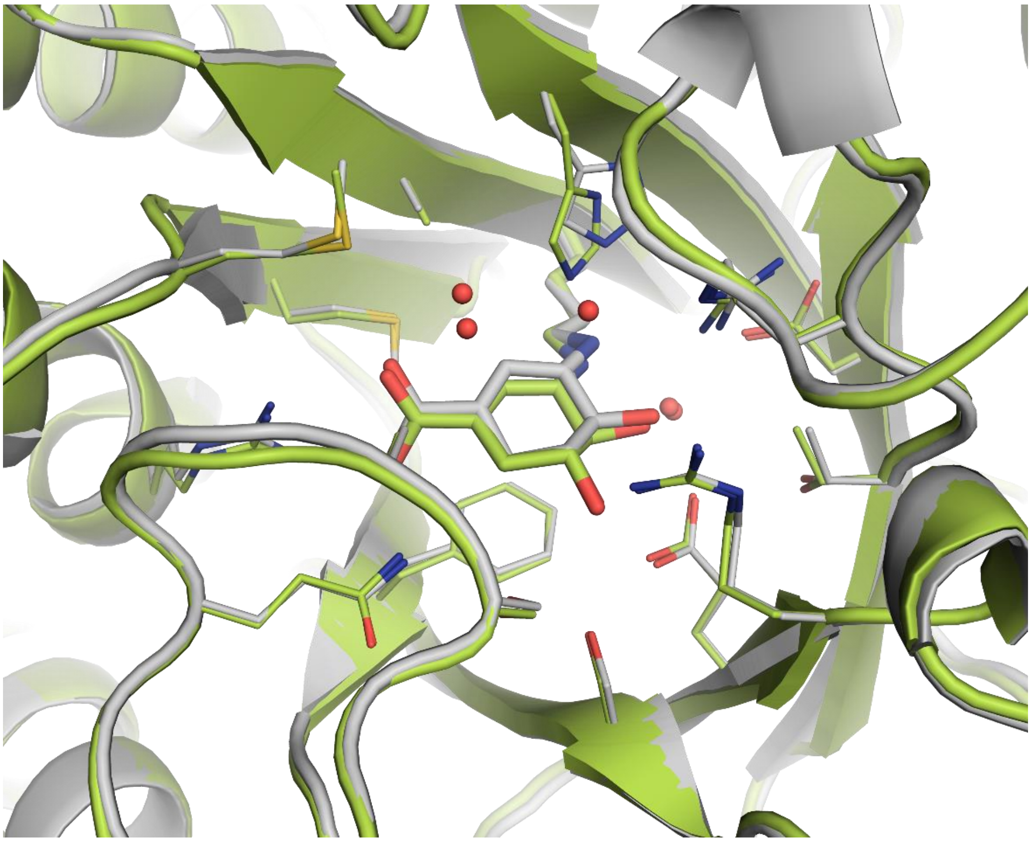

Figure S2. Superposition of the crystal structure of *St*-DHQ1 covalently modified by hydroxylamine **6** (green, PDB ID 8B2B, 1.90 Å, chain A) with of the trapped enzyme-product Schiff base intermediate obtained by reduction with sodium borohydride (gray, PDB ID 1QFE, 2.10 Å, chain A). The rms value between chains is 0.3.

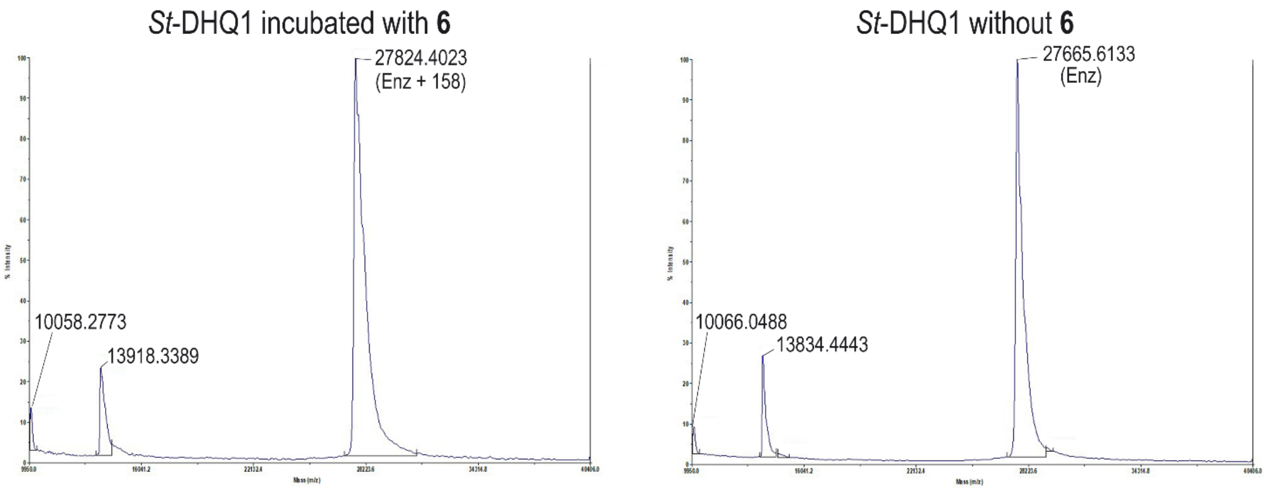

Figure S3. MALDI spectra of *St*-DHQ1 after incubation with 3-hydroxylaminoquinic acid (**6**) (left) and the control (right). Incubation conditions were potassium phosphate buffer (50 mM, pH 7.0), 25 °C and (1:120) protein/ligand ratio, 24 h. After incubation and for MALDI analysis, the samples were successively washed with 5 mM sodium bicarbonate by centrifugation at 4 ºC using Amicon® centrifugal filters and concentrated. Sinapic acid was used as a matrix.

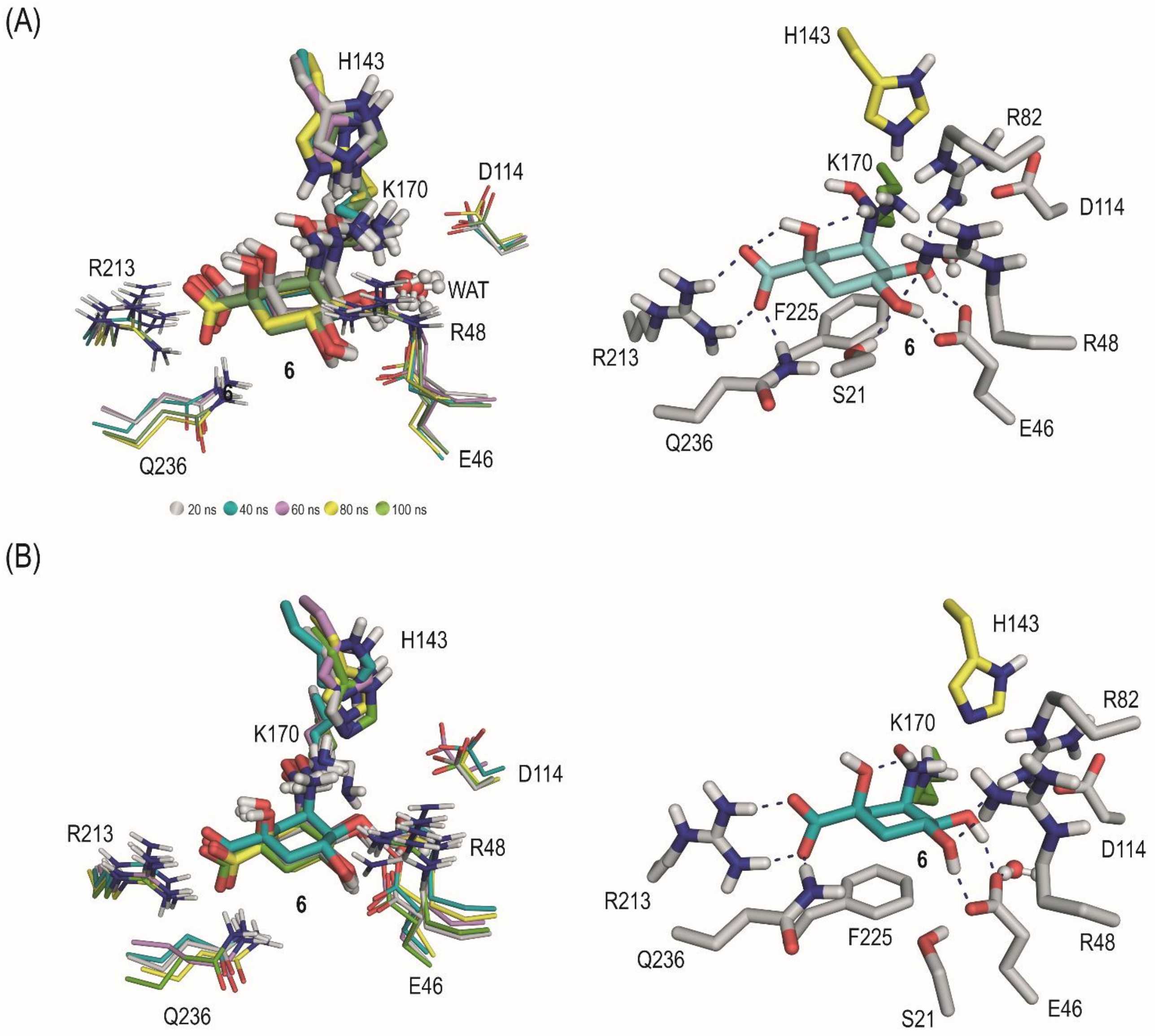

Figure S4. Comparison of several snapshots of the *Sa*-DHQ1/**6** binary complexes during 100 ns of MD simulation in which the two plausible protonation states of the NHOH moiety in **6** [neutral (A) and protonated (B)] and residue H143 [dual (A) and neutral (B)] were considered. Detailed view of the main contacts of **6** with the residues of the active site are also provided (right) as blue dashed lines. Relevant residues side chains are shown and labeled. The catalytic residues and the ligand are highlighted as sticks in the overlapping drawing. For both cases, note how the catalytic lysine residue is capable to reach the C3 position in **6**.

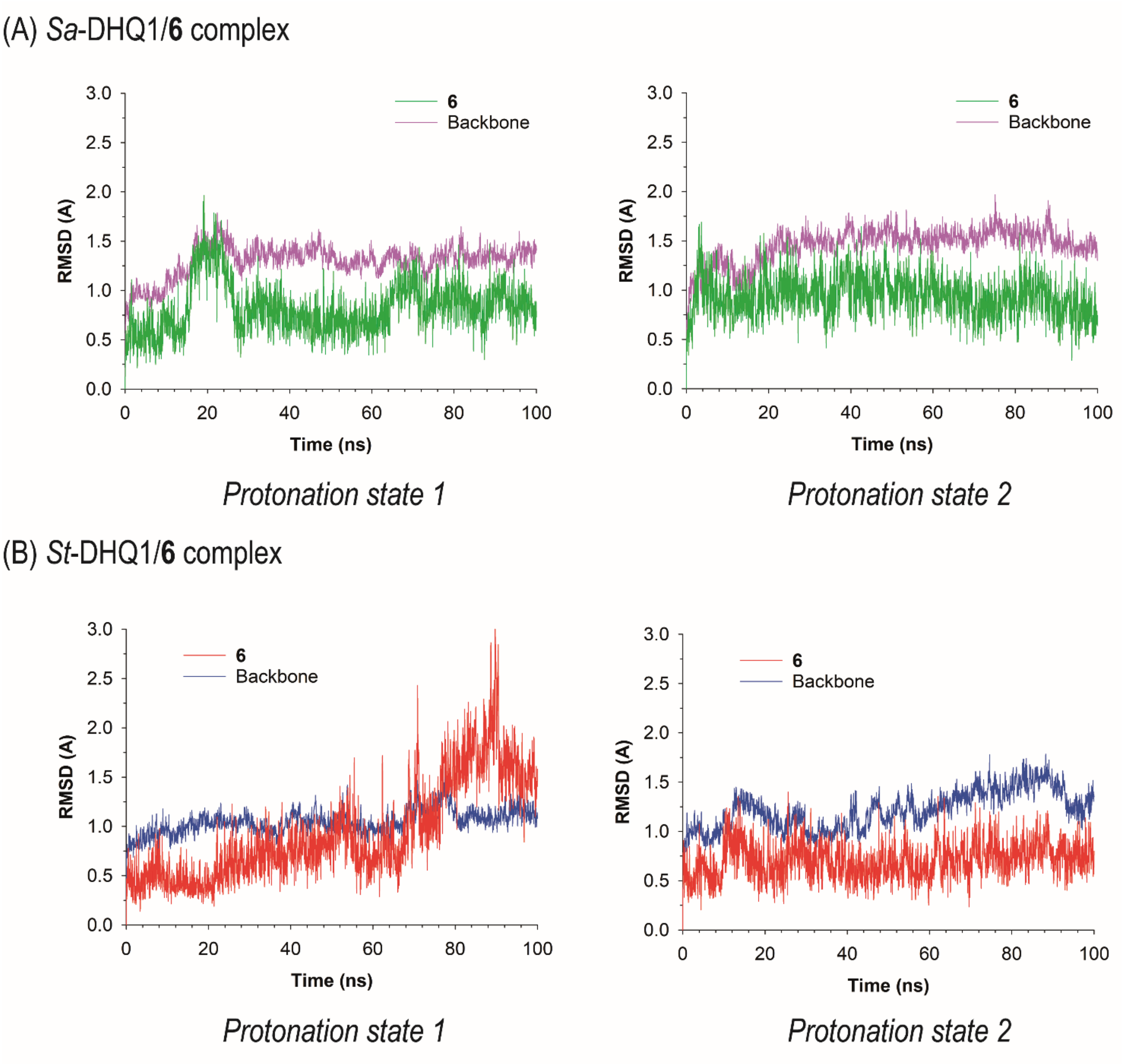

Figure S5. RMSD plots for the protein backbone (Cα, C, O and N atoms) and the ligands calculated from the MD simulations of the complexes: (A) *Sa*-DHQ1/**6** and (B) *St*-DHQ1/**6**; in which the two plausible protonation states of the NHOH moiety in **6** [neutral (left) and protonated (right); protonation state 1] and histidine residue [(dual (left) and neutral (right); protonation state 2] were considered. Note the low rmsd values of the protein backbone as well as the ligand. Both facts reveal the high stability of the enzyme complexes as well as of the ligand within the binding pocket. Average values for the protein backbone and compound **6** ranking between 1.0–1.4 Å and 0.8–0.9 Å, respectively, were obtained.

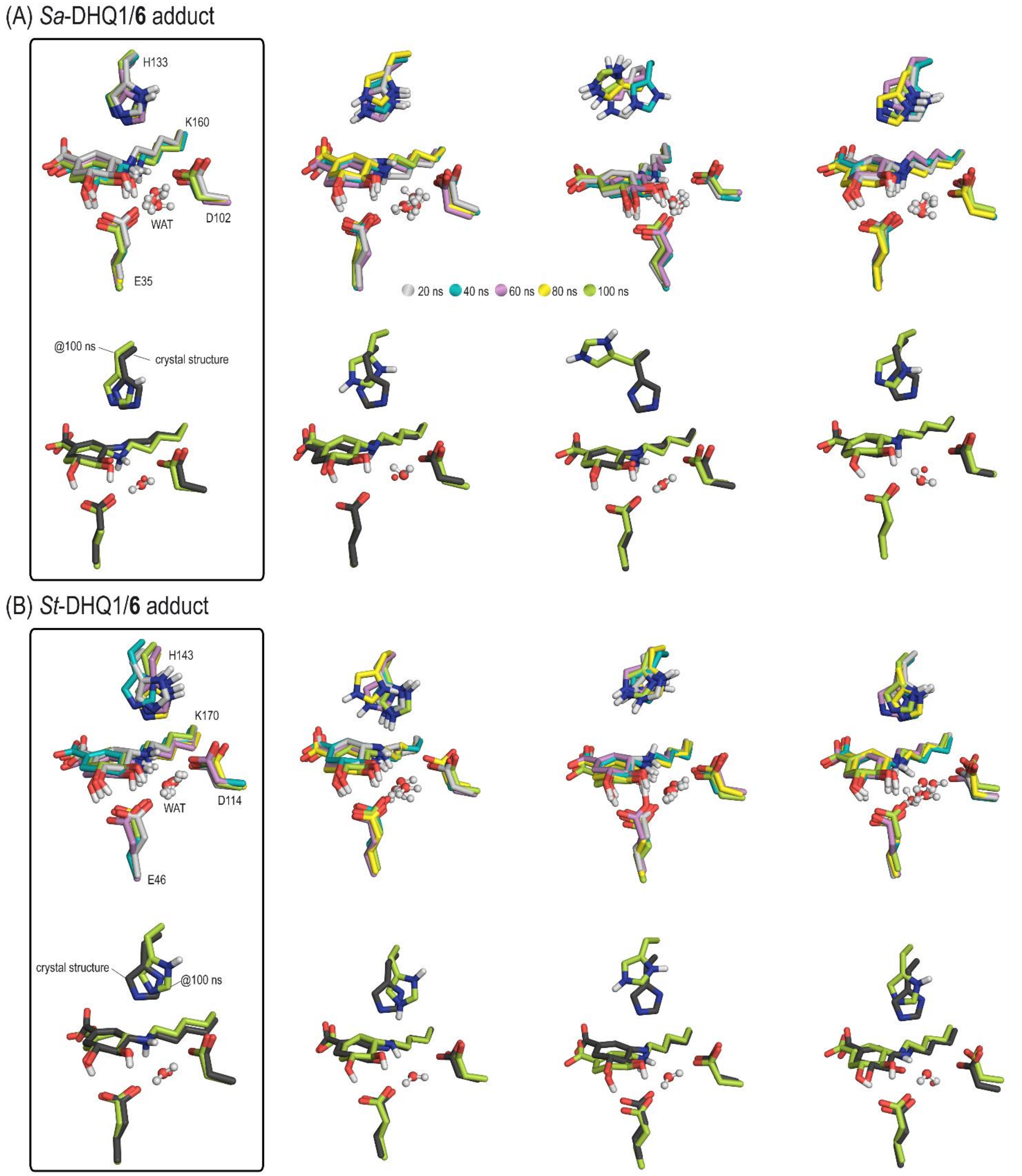

Figure S6. Analysis by MD simulation studies of the protonation state of the modified catalytic lysine and histidine residues in the herein reported crystal structures *Sa*-DHQ1/**6** (A) and *St*-DHQ1/**6** (B). Comparison of several snapshots of the *Sa*-DHQ1/**6** (A) and *St*-DHQ1/**6** (B) adducts during 100 ns of MD simulation and superposition of the snapshot at 100 ns and the solved crystal structures are provided. Relevant residues side chains are shown and labeled. Note how when H133/H143 are considered dual large motion or displacement from the location obtained was obtained. In addition, when these residues and the modified K160/K170 were used in their neutral forms instability in the pocket composed by D102/D114 and the structural water molecule was observed. The more suitable protonation state for both catalytic residues are highlighted with a black box.

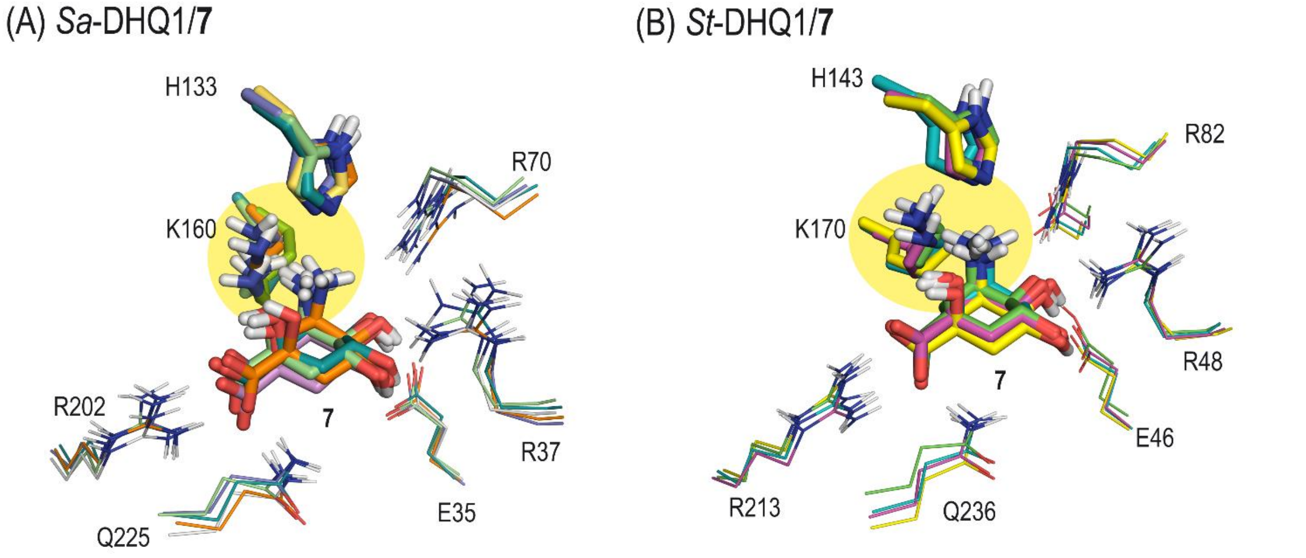

Figure S7. Comparison of several snapshots of the *Sa*-DHQ1/**7** (A) and *St*-DHQ1/**7** (B) binary complexes during 100 ns of MD simulation. Relevant residues side chains are shown and labeled. The catalytic residues and the ligand are highlighted as sticks. Note how the position of the catalytic lysine residue (yellow shadow) is frozen nearby the C2 binding pocket by hydrogen-bonding interaction with the ammonium group in **7**.

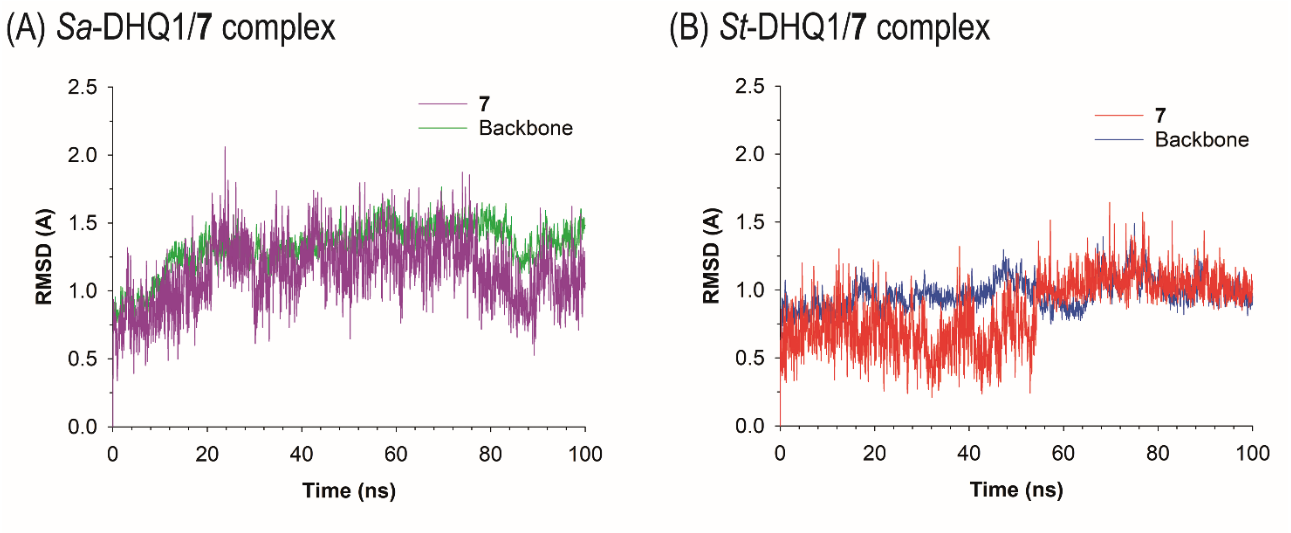

Figure S8. RMSD plots for the protein backbone (Cα, C, O and N atoms) and the ligands calculated from the MD simulations of the complexes: (A) *Sa*-DHQ1/**7** and (B) *St*-DHQ1/**7**. The catalytic histidine residue was considered in its neutral form and the ligand as ammonium salt. Note the low rmsd values of the protein backbone as well as the ligand. Average values for the protein backbone and compound **7** ranking between 1.0–1.3 Å and 0.8–1.1 Å, respectively, were obtained.

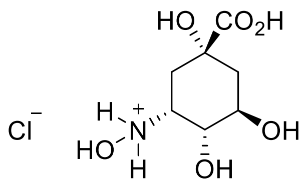

6

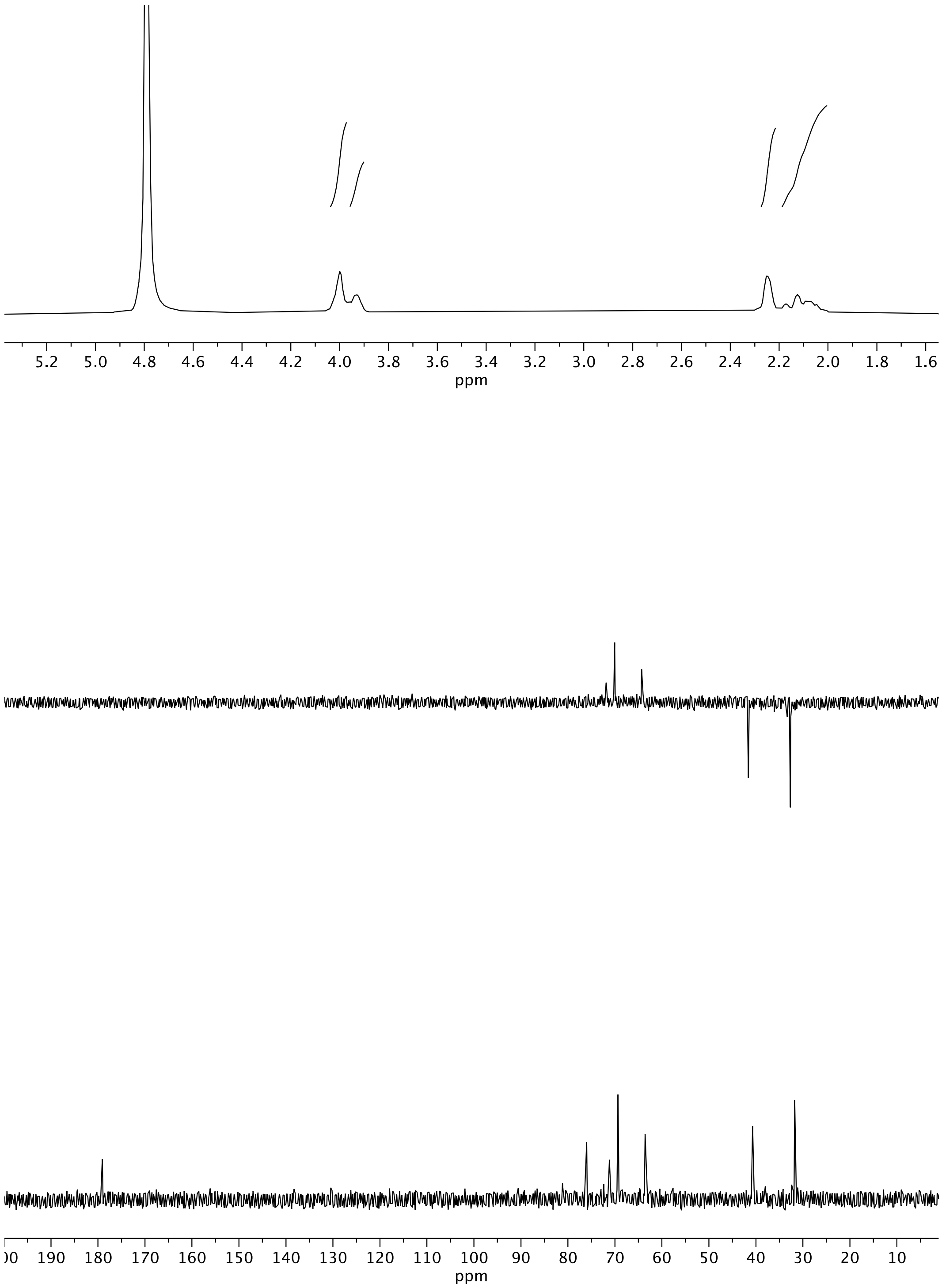

Figure S9. NMR spectra for compound **6**

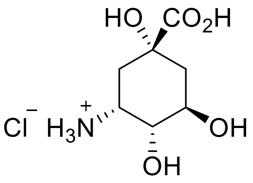

7

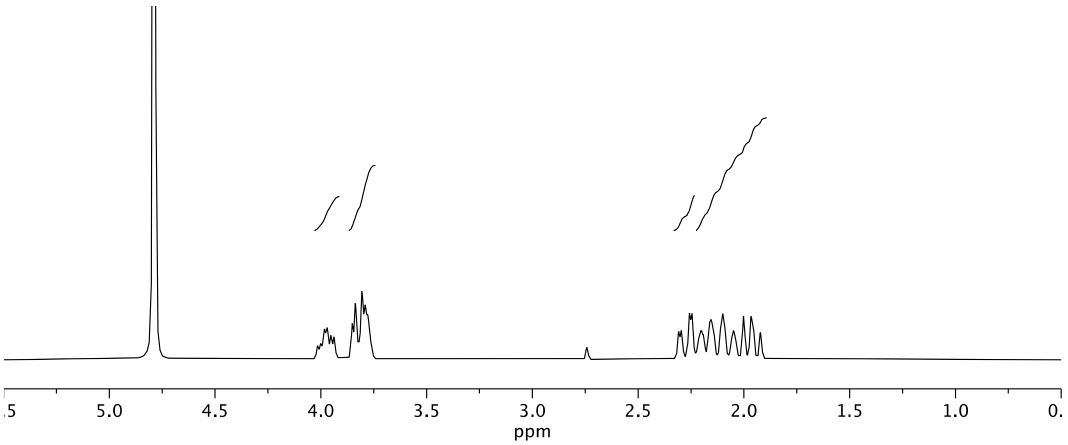

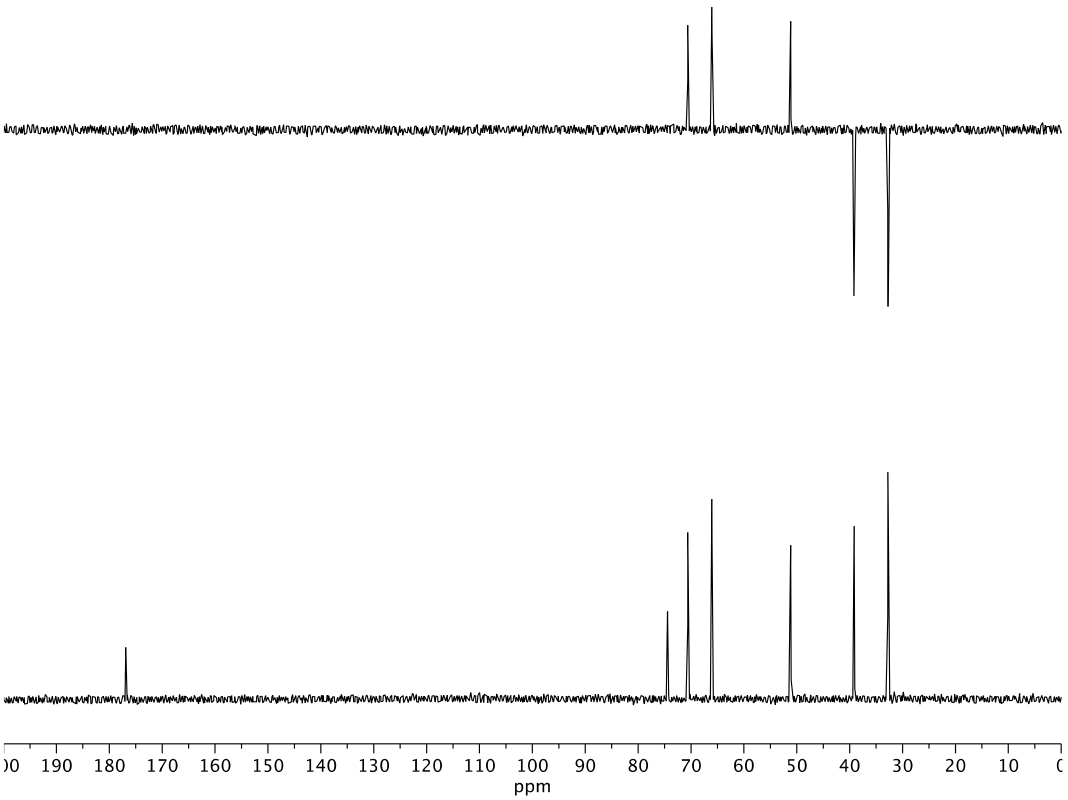

Figure S10. NMR spectra for compound **7**

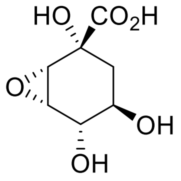

8

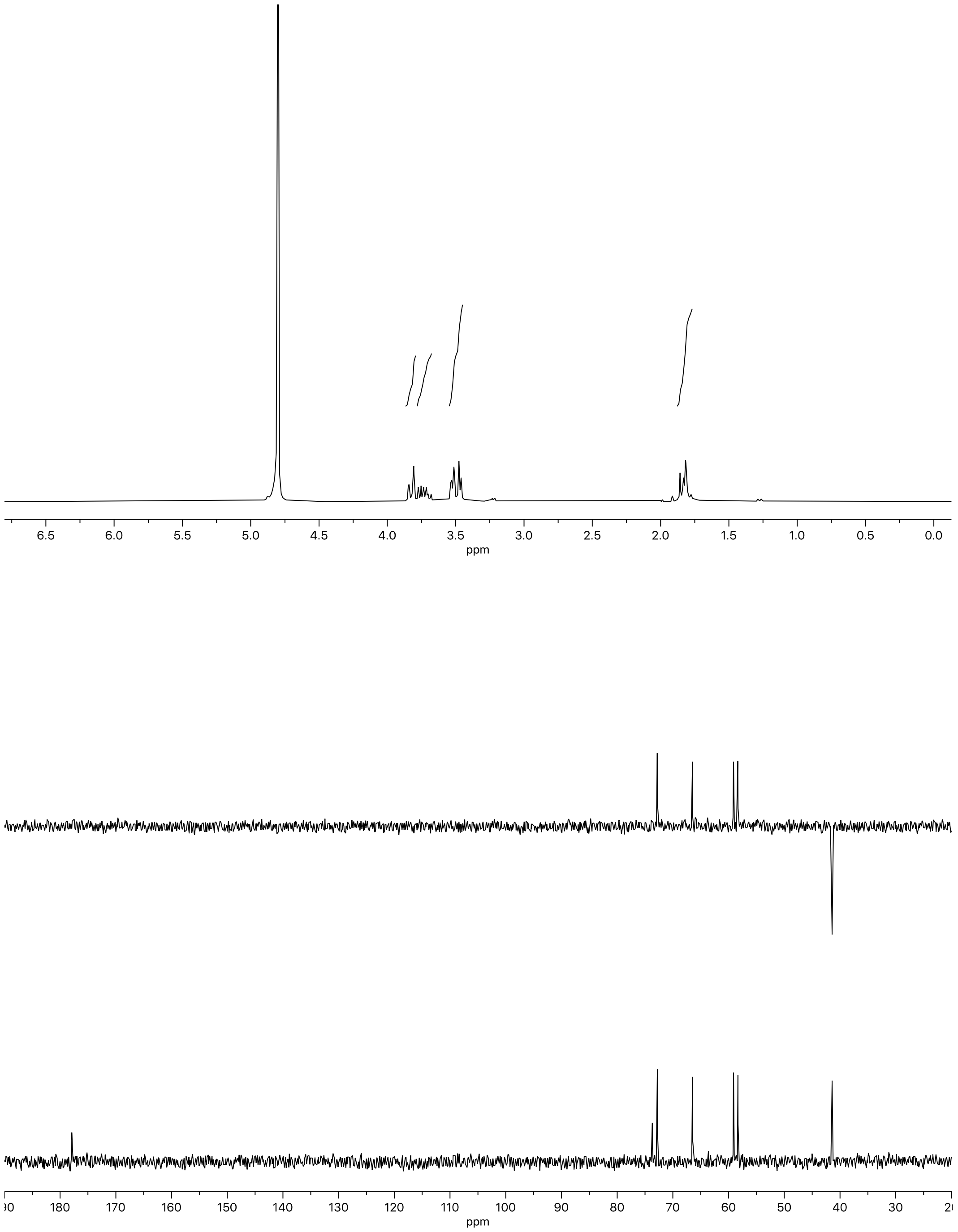

Figure S11. NMR spectra for compound **8**
